# Co‐Self‐Assembled Interface Engineering Assisted for Bend‐Resistant and Efficient Flexible Perovskite Solar Cells

**DOI:** 10.1002/advs.202509724

**Published:** 2025-10-24

**Authors:** Chunlong Wang, Chu Zhang, Qingxue Wang, Hao Li, Yutong Wu, Yue Zhao, Shennan Chen, Liang Li, Mingjun Nie, Jiaxing Song, Zaifang Li, Yonggang Yu, Lei Shi, Yongchun Ye, Yu Wang, Tingli Ma, Wensheng Yan

**Affiliations:** ^1^ New Energy Materials and Devices Laboratory College of Materials and Chemistry China Jiliang University (CJLU) Hangzhou 310018 China; ^2^ Key Laboratory of Intelligent Manufacturing Quality Big Data Tracing and Analysis of Zhejiang Province College of Science China Jiliang University (CJLU) Hangzhou 310018 China; ^3^ College of optical and electronic technology China Jiliang University (CJLU) Hangzhou 310018 China; ^4^ Hangzhou Keneng New Energy Co., Ltd. No. 2728, Jiangdong Second Road, Hezhuang Street, Qiantang District Hangzhou Zhejiang 310018 China; ^5^ Zhejiang Key Laboratory of Advanced Tandem Photovoltaic Technology College of Biological and Chemical Engineering Jiaxing University Jiaxing 314001 China; ^6^ Hangzhou Zhongneng Photoelectricity Technology Co., Ltd. 22nd Avenue, Qiantang New Area Hangzhou Zhejiang 310018 China; ^7^ Graduate School of Life Science and Systems Engineering Kyushu Institute of Technology (KIT) Fukuoka 804–8550 Japan; ^8^ Institute of Carbon Neutrality and New Energy School of Electronics and Information Hangzhou Dianzi University (HDU) Hangzhou 310018 China

**Keywords:** 4‐nitrophenyl phosphate, defect passivation, flexible perovskite solar cells, self‐assembly monolayer, stability

## Abstract

Flexible perovskite solar cells (F‐PSCs) have attracted considerable interest for their superior mechanical flexibility. Nonetheless, cryptic bottom‐interface defects hinder further improvements in device performance. Here, a co‐self‐assembled monolayer (Co‐SAM) engineering strategy is implemented by integrating 4‐nitrophenyl phosphate (PNPP) into [4‐(3,6‐dimethyl‐9H‐carbazol‐9‐yl) butyl] phosphonic acid (Me‐4PACz) to improve the NiO_x_/perovskite (PVK) interface. This technique enhanced the surface uniformity and hydrophilic nature of the NiO_x_/Me‐4PACz, while promoting favorable growth of PVK crystal orientation. Furthermore, the PNPP effectively mitigates the generation of defects at the NiO_x_ surface and the underlying PVK, ultimately significantly improving the interfacial charge transfer efficiency. Consequently, the efficiency of F‐PSCs rose from 21.46% to 23.66%. Due to better stress distribution within the PVK and stronger adhesion at the NiO_x_/PVK boundary, the F‐PSCs retained 80% of their original efficiency even after undergoing 10 000 bending cycles. Notably, PNPP exhibited an outstanding capacity to capture PbI_2_, contributing to the potential for reducing Pb leakage of the device under operational conditions.

## Graphical abstract

In this study, by incorporating 4‐nitrophenyl phosphate (PNPP) into [4‐(3,6‐dimethyl‐9H‐carbazol‐9‐yl)butyl]phosphonic acid (Me‐4PACz), an efficient co‐assembled monolayer (Co‐SAM) is constructed to modulate the bottom interface region, thereby developing highly efficient and stable inverted flexible perovskite solar cells. As a result, we achieved a champion device efficiency of 23.66%, and the device retained 80% of its initial efficiency after 10 000 continuous bending cycles under one‐sun illumination.

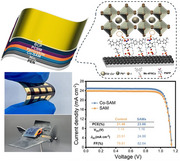

## 1 Introduction

Flexible perovskite solar cells exhibit significant potential for integration into wearable devices, building‐integrated photovoltaics (BIPV), and mobile energy sources due to their higher power conversion efficiency (PCE), cost‐effective fabrication, and remarkable mechanical flexibility. Currently, small‐area single‐junction F‐PSCs have achieved a PCE of up to 26.61%. Despite this progress, F‐PSCs still exhibit lower efficiency compared with rigid variants, and their operational durability remains insufficient for widespread practical use. Inverted perovskite solar cells (IPSCs), with enhanced stability and compatibility with low‐temperature solution‐based processes, have emerged as a promising architecture for high‐performance F‐PSCs development. Among the various materials used, NiO_x_ has become a common choice for the hole transport layer (HTL) in both inverted and tandem cell designs, offering advantages such as affordability, robust stability, and scalability. However, the presence of interfacial defects such as vacancy‐oxygen (VO) defects, uncoordinated nickel ions (Ni^+^), and lead ions (Pb^2+^) at the NiO_x_ surface and the bottom of the perovskite layer contributes to intense nonradiative recombination. Additionally, detrimental interfacial interactions between NiO_x_ and perovskite layers amplify charge‐carrier losses and severely reduce the voltage open circuit (*V*_OC_). These interfacial challenges remain key barriers to the realization of highly efficient and durable inverted F‐PSCs devices.

In contrast to the relatively simple modulation of top‐interface defects in perovskite solar cells, adjusting the characteristics of the bottom interface presents significantly greater complexity and technical challenges. To overcome the interfacial limitations at NiO_x_/perovskite junctions, diverse strategies involving interface modification and defect passivation have been developed. These include the use of various functional additives, such as inorganic salts, organic molecules, self‐assembled monolayers (SAMs), and 2D materials introduced at the heterojunction. Among these, SAMs have emerged as a particularly effective solution due to their strong capabilities in energy level modulation and adjusting interfacial properties. For instance, Li et al. demonstrated that NiO_x_ nanocrystals modified with SAMs could substantially enhance the interfacial quality at the NiO_x_/perovskite junction, resulting in suppressed charge recombination and improved hole extraction.^[^ ^1^ ^]^ This led to a notable efficiency of 24.7% (certified 24.4%) in flexible tandem perovskite solar cells. In another study, Yang et al. incorporated [4‐(3,6‐dimethyl‐9H‐carbazol‐9‐yl) butyl] phosphonic acid (Me‐4PACz) as a SAM passivator, which effectively facilitated charge extraction and transport, achieving a peak efficiency of 23.29%.^[^ ^2^ ^]^ Despite the demonstrated versatility and performance of Me‐4PACz, its molecular design featuring methyl substituents results in increased steric hindrance and strong hydrophobicity. This structural nature impedes the formation of uniform perovskite layers on its surface post‐deposition, limiting its ability to simultaneously improve NiO_x_/perovskite interfacial characteristics and facilitate dense perovskite film formation in F‐PSCs. To address these limitations, Liu et al. introduced a Co‐SAM composed of 4′,4′,4′’‐nitrilotribenzoic acid and Me‐4PACz at the buried interface of inverted perovskite solar cells. This design significantly enhanced charge transport across the interface and improved the quality of the perovskite film, leading to a certified power conversion efficiency (PCE) of 26.54%.^[^ ^3^ ^]^ Similarly, Sargent et al. employed a Co‐SAM strategy using 3‐sulfanylpropanoic acid combined with 2‐(3,6‐Dibromo‐9H‐carbazol‐9‐yl) ethyl] phosphonic acid (2PACz), effectively reducing molecular aggregation and expanding substrate coverage, while modulating film defects contributing to a device efficiency of 25.3%.^[^ ^4^ ^]^ The development and application of novel SAM molecules have proven effective in enhancing perovskite film uniformity and device efficiency. Replacing methyl groups in Me‐4PACz with glycol monomethyl ether (GM) chains, for example, resulted in a SAM variant ([4‐(3,6‐glycol monomethyl ether‐9H‐carbazol‐9‐yl) butyl] phosphonic acid) with enhanced hydrophilic properties.^[^ ^5^ ^]^ Furthermore, new amphiphilic SAMs have shown promise in promoting better perovskite precursor spreading. Nonetheless, SAM molecules inherently tend to cluster during deposition, which undermines the formation of smooth, compact monolayers. Consequently, the integration of Co‐SAM strategies is more effective for optimizing molecular adsorption on substrate surfaces and exerting greater control over critical perovskite film parameters such as bottom‐interface defect density and internal stress distribution. This approach offers significant advantages in advancing the performance and stability of next‐generation F‐PSCs.

**Figure 1:**
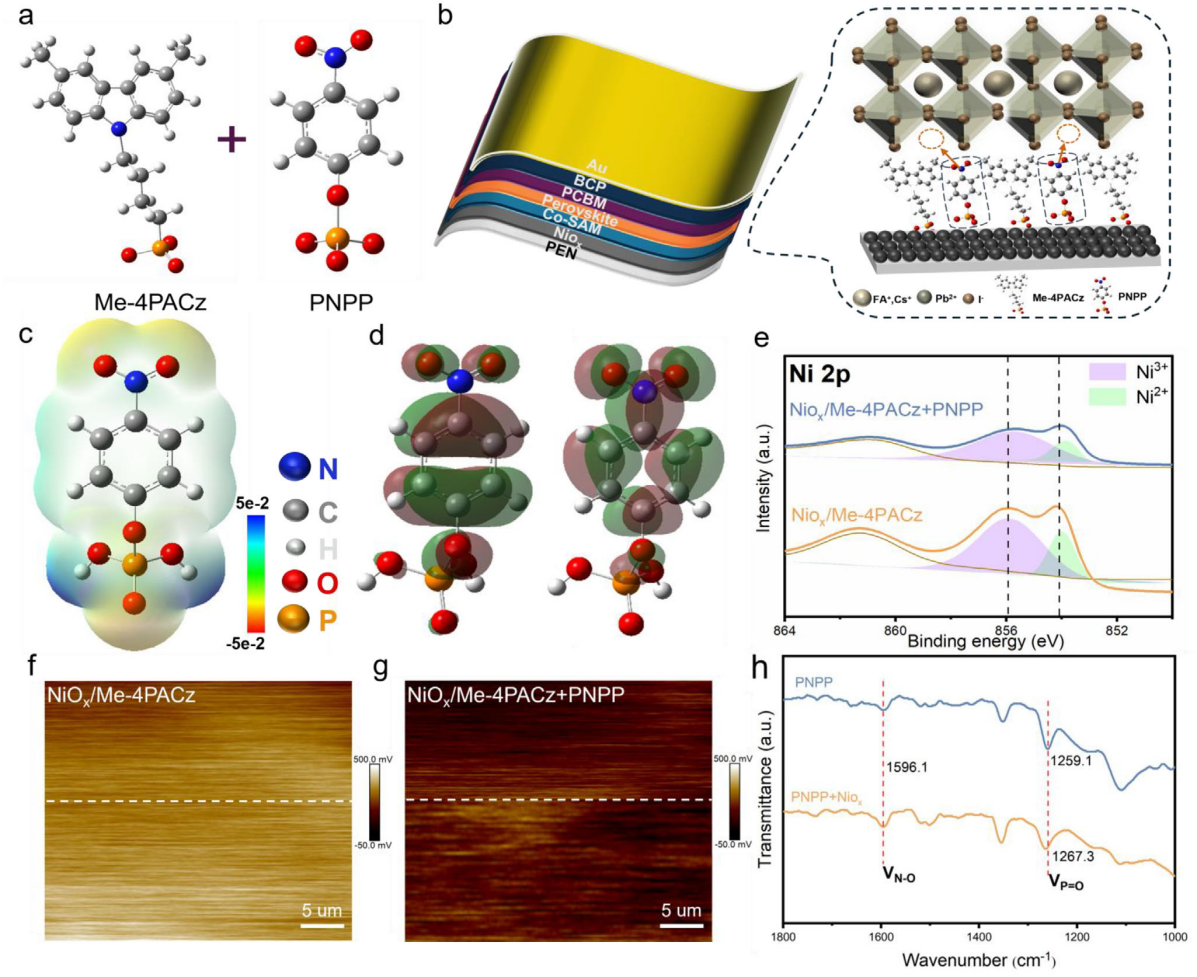
a) Molecular structures of Me‐4PACz and PNPP. b) Schematic diagram of the inverted device structure and the interaction role of Co‐SAM with NiO_x_ and perovskite films. c) Gaussian calculated the electrostatic potential of the PNPP molecule. d) The corresponding electronic cloudy distribution of PNPP. e) XPS of NiO_x_/Me‐4PACz+PNPP, NiO_x_/Me‐4PACz. f,g) KPFM images of NiO_x_/Me‐4PACz and NiO_x_/Me‐4PACz+PNPP. (h) FTIR of PNPP+NiO_x_ and PNPP.

In this study, the Co‐SAM strategy was further advanced by PNPP into Me‐4PACz to enhance the NiO_x_/PVK interface in F‐PSCs (**Figure** **1**a), resulting in improved PCE and device stability. The introduction of PNPP effectively increases the surface hydrophilicity and film‐forming uniformity of the NiO_x_/Me‐4PACz layer. Importantly, the functional groups in PNPP, namely P═O and N─O moieties, engage in targeted interactions with uncoordinated metal ion defects at the NiO_x_ surface and the perovskite's bottom interface. Through first‐principles density functional theory (DFT) calculations, PNPP retains a strong binding affinity for PVK while interacting with NiO_x_ (─P═O─Ni^2+^), with a binding energy of ‐0.549 eV (─N─O‐Pb^2+^). These interactions enable simultaneous passivation of interfacial defects, thereby mitigating non‐radiative recombination pathways. The modified NiO_x_/Me‐4PACz+PNPP interface also exhibits improved energy‐level alignment, facilitating more efficient charge extraction. Additionally, the perovskite films deposited on this surface show enhanced crystalline orientation, contributing to superior charge transport characteristics. Ultimately, the PCE of the inverted F‐PSCs increased from 21.46% to 23.66%. The rigid champion device achieved an efficiency of 24.80%. Furthermore, through Co‐SAM modification, scalable large‐area rigid modules and flexible modules achieved efficiencies of 17.01% and 15.72%, respectively, on an active area of 52.8 cm^2^. Meanwhile, the devices demonstrated substantial mechanical flexibility and long‐term operational durability. The reduction in residual stress within the perovskite layer and the strengthened interfacial adhesion allowed the F‐PSCs to retain 80% of their initial efficiency after undergoing 10 000 continuous bending cycles. Notably, PNNP exhibited an outstanding capacity to capture PbI_2_, underscoring the effectiveness of the PNPP‐enhanced Co‐SAM approach in advancing robust, high‐efficiency F‐PSCs for practical applications.

## 2 Results and Discussion

To explore the benefits of the Co‐SAM approach, a representative inverted F‐PSCs model was constructed, and the molecular interactions at the interface were schematically illustrated (Figure 1b). When Me‐4PACz is applied alone, its bulky molecular structure hinders the formation of a densely packed monolayer on the NiO_x_ surface due to steric effects.^[^ ^6^ ^]^ However, introducing PNPP to create a Co‐SAM structure effectively fills the voids between Me‐4PACz molecules, significantly enhancing the uniformity and surface coverage of the modified layer. To experimentally validate this mechanism, contact angle measurements were carried out to assess the wetting behavior of perovskite precursor solutions on various substrates.^[^ ^7^ ^]^ On bare NiO_x_, the average contact angle was 18°, while the Me‐4PACz‐modified surface exhibited an increased contact angle of 28°, which correlates with the observed poor uniformity in the resulting perovskite films. This observation aligns with previous studies reporting that Me‐4PACz resists wetting by polar precursor solutions, adversely affecting film formation.^[^ ^8^ ^]^ After modifying the surface with the Co‐SAM, the contact angle decreased markedly to 12° (Figure S1, Supporting Information), and the perovskite film uniformity improved substantially (Figure S2, Supporting Information). Meanwhile, a smaller contact angle results in reduced Gibbs free energy for heterogeneous nucleation, thereby assisting the nucleation process. Higher nucleation density will promote the film densification process.^[^ ^9^ ^]^ This improvement primarily originates from the presence of the polar nitro (─N═O) functional group in PNPP, which can form hydrogen bonds with polar solvents such as DMF, thereby facilitating better precursor spreading and film coverage.^[^ ^10^ ^]^

To examine the influence of the Co‐SAM strategy on the surface characteristics of NiO_x_, atomic force microscopy (AFM) measurements were conducted. The analysis revealed that the NiO_x_ substrate treated with the combined Me‐4PACz+PNPP interface modifier exhibited a notably reduced surface roughness of 4.36 nm, in comparison to the 6.59 nm observed for the substrate modified with Me‐4PACz alone.^[^ ^11^ ^]^ In addition, the topographical height distribution was more uniform (Figure S3, Supporting Information), suggesting that the Co‐SAM treatment significantly smooths the NiO_x_ surface and mitigates height fluctuations. This improvement can enhance the interfacial contact between the NiO_x_ layer and the perovskite, thereby facilitating the subsequent crystallization of the perovskite,^[^ ^12^ ^]^ as evidenced by the data in Figure S4, (Supporting Information). Furthermore, the phosphate moieties present in PNPP molecules provide additional chemical functionality that strengthens the interfacial defect passivation effect of Me‐4PACz on the NiO_x_ surface.^[^ ^13^ ^]^

To further explore the chemical interactions between PNPP in Co‐SAM and NiO_x_, Gaussian calculations were conducted to analyze the electrostatic potential distribution of PNPP (Figure 1c, Supporting Information). The results indicate that the phosphate groups within the molecule are negatively charged, signifying their electron‐donating nature. This characteristic helps reduce charge recombination, thereby enhancing the *V*_OC_ of the solar cell. In contrast, the nitro groups exhibit a positive charge, and their interaction with either the electron transport layer or the hole transport layer facilitates improved carrier extraction and transport efficiency.^[^ ^14^ ^]^ Additionally, the highest occupied molecular orbital (HOMO) and lowest unoccupied molecular orbital (LUMO) energy levels of PNPP were computed (Figure 1d, Supporting Information), revealing a HOMO level of 0.039 eV and a LUMO level of −0.380 eV, with an energy gap of 0.419 eV. The narrow bandgap suggests that PNPP is prone to hole donation, which contributes to enhanced carrier transport properties within the device.^[^ ^15^ ^]^

The surface contact potential difference (CPD) of NiO_x_/Me‐4PACz and NiO_x_/Me‐4PACz+PNPP thin films was examined using Kelvin probe force microscopy (KPFM). As presented in Figure 1f,g, the NiO_x_/Me‐4PACz+PNPP film exhibited a significantly lower CPD compared to the NiO_x_/Me‐4PACz film. This reduction in CPD signifies a downward shift of the Fermi level (EF) in the Me‐4PACz layer, a conclusion further validated by ultraviolet photoelectron spectroscopy (UPS).^[^ ^16^ ^]^ Moreover, the corresponding surface potential distribution analysis verified that the Co‐SAM modification strategy resulted in a more uniform electric field distribution (Figure S5, Supporting Information). This systematic enhancement establishes Co‐SAM as a robust approach for simultaneously boosting device performance and operational stability.^[^ ^17^ ^]^ To assess the chemical interactions at the NiO_x_/perovskite interface in the presence of Co‐SAM, x‐ray photoelectron spectroscopy (XPS) analysis was conducted. Figure 1e displays the Ni 2*p* core‐level spectra of NiO_x_, where the binding energies for Ni^2+^ and Ni^3+^ are located at 853.8 and 855.9 eV (Table S4, Supporting Information), respectively. Following the introduction of Co‐SAM, a noticeable shift was observed in the Ni^3+^ binding energy, implying an electrostatic interaction between PNPP and NiO_x_.^[^ ^18^ ^]^ Additionally, the Ni^3+^/Ni^2+^ ratio increased from 2.76 to 3.81, indicating an enhancement in the electrical conductivity of the NiO_x_/Co‐SAM layer. This conclusion is further supported by current–voltage (I–V) measurements of ITO/NiO_x_/Me‐4PACz/Ag and NiO_x_/Me‐4PACz+PNPP/Ag configurations (Figure S6 and Table S1, Supporting Information). Moreover, the observed shift of Ni ion binding energies to lower values on the NiO_x_/Co‐SAM surface confirmed the interaction between the phosphate groups in PNPP and NiO_x_. Further insights were gained from the nitrogen content and the emergence of additional valence states detected on the NiO_x_/Me‐4PACz+PNPP surface (Figure S7, Supporting Information), suggesting the co‐existence of both Me‐4PACz and PNPP molecules.^[^ ^19^ ^]^ Analysis via Fourier transform infrared spectroscopy (FTIR) (Figure 1h) indicated no significant change in the vibrational frequency of the N‐O bond following PNPP interaction with NiO_x_. However, a redshift of 8.2 cm^−1^ was observed in the P═O bond vibrational frequency relative to pure PNPP, indicating a chelation interaction between the phosphate group and the NiO_x_ surface.^[^ ^20^ ^]^ To further confirm this interaction, X‐ray diffraction (XRD) measurements were carried out for NiO_x_, PNPP, and NiO_x_/PNPP samples. NiO_x_ displayed characteristic diffraction peaks corresponding to the (111) and (200) planes (Figure S7, Supporting Information). Upon the addition of PNPP, the (200) peak exhibited a blue shift, suggesting that PNPP incorporation influenced the lattice parameters and crystallite size of NiO_x_, indicative of lattice expansion.^[^ ^21^ ^]^ Moreover, the downfield shift observed in the nuclear magnetic resonance ^31^P (NMR) (nuclear magnetic resonance) spectrum after mixing PNPP with NiO_x_ fundamentally originates from the decreased electron density around the phosphorus atom (Figure S8, Supporting Information). This occurs when the oxygen atom of the phosphate group (─OPO_3_^2−^) in PNPP coordinates with the Ni^2+^ sites on the NiO_x_ surface, forming an O→Ni coordination bond. Acting as a Lewis acid, the Ni^2+^ ion withdraws electron density from the oxygen atom of the phosphate group. This deshielding effect is transmitted through the P─O bond to the central phosphorus atom, reducing the shielding effect of the electron cloud around the phosphorus nucleus during NMR measurements. As a result, resonance occurs at a lower external magnetic field, corresponding to an increase in the chemical shift (δ value). This shift provides direct evidence that the phosphate group is involved in coordination, serving as structural proof of a chemical interaction between the two components.^[^ ^22^ ^]^ Collectively, these findings confirm that the phosphate groups within PNPP molecules engage in chelation interactions with the NiO_x_ surface.

**Figure 2:**
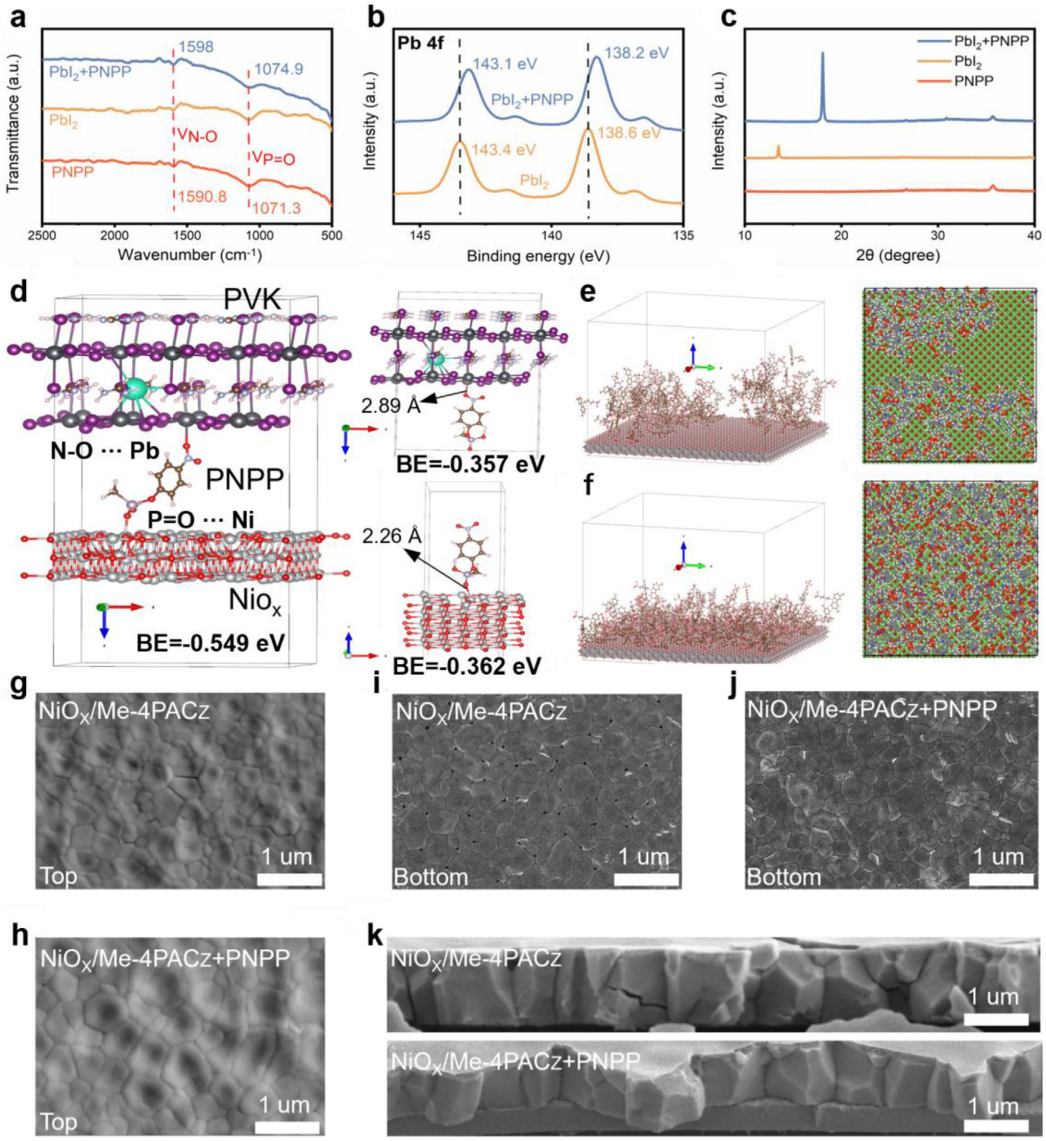
a) FTIR of PNPP+PbI_2_, PbI_2_, and PNPP. b) XPS of Pb 4f for PNPP+PbI_2_ and PbI_2_. c) XRD of PbI_2_+PNPP, PbI_2_, and PNPP. d) The rational design of molecules through DFT calculations and corresponding binding models of PVK, PNPP with NiO_x_ surface. e) DFT‐optimized structure of Me‐4PACz on NiO_x_ surface. f) DFT‐optimized structure of Me‐4PACz+PNPP on NiO_x_ surface. Where the pulper, red, dark pink, light pink, brown, silver, black, purple, and green atoms respectively represent the O, P, H, C, N, Pb, I, and Cs atoms. g,k) Top‐view SEM images from the buried interface and surface of perovskite films and cross‐sectional SEM images based on NiO_x_/Me‐4PACz and NiO_x_/Co‐SAM films.

To analyze the interaction mechanism between PNPP within Co‐SAM and the buried interface at the bottom of the perovskite layer, a combination of analytical techniques, FTIR, XPS, and XRD, was utilized.^[^ ^23^ ^]^ FTIR analysis indicated that, upon mixing PNPP with PbI_2_, the characteristic vibrational frequencies associated with the P═O and N─O bonds underwent blueshifts of 3.7 and 8.8 cm^−1^, respectively (**Figure** **2**a), suggesting that these functional groups engage in interactions with Pb^2+^ ions at the buried bottom interface of the perovskite.^[^ ^24^ ^]^ XPS measurements further supported this finding: in the PNPP‐PbI_2_ composite, the binding energies of Pb and I shifted toward lower values compared to those in pure PbI_2_ (Figure 2b; Figure S9 and Table S5, Supporting Information), while the O binding energy exhibited an upward shift (Figure S9, Supporting Information). These results indicate the presence of a Lewis acid‐base interaction, with nitro groups in PNPP coordinating with Pb^2+^ species.^[^ ^2^ ^]^ XRD characterization provided additional structural insights. Upon incorporation of PNPP into PbI_2_, the diffraction pattern reflected the formation of a new crystalline phase distinct from either pure PbI_2_ or PNPP alone, a low‐dimensional PNPP, PbI_2_ complex (Figure 2c). Considering this, along with the earlier evidence of phosphate group chelation with the NiO_x_ surface, it can be deduced that the unbound nitro groups in PNPP are likely involved in passivating undercoordinated Pb defects located at the bottom surface of the perovskite film.^[^ ^25^ ^] 1^H NMR spectroscopy further confirmed the strong chemical coordination between PNPP and the PbI_2_ framework within the range of 7–8.4 ppm (Figure S10, Supporting Information). This observation suggests that Pb^2+^ is likely to coordinate with the oxygen atom of the nitro group (─NO_2_), possibly forming an O→Pb coordination bond. This interaction significantly enhances the electron‐withdrawing capability of the nitro group, thereby considerably reducing the electron density of the ortho hydrogen (Hα) and resulting in a characteristic downfield shift of its NMR signal.^[^ ^26^ ^]^

Based on first‐principles density functional theory (DFT) calculations, we initially computed the binding energies between PNPP and PVK, as well as between PNPP and NiO_x_, obtaining values of ─N─O─Pb^2+^ and ─P═O─Ni^2+^ are −0.357and 0.362 eV (Figure 2d), respectively. Subsequently, we further evaluated the binding energy of PNPP with PVK in the presence of simultaneous interaction with NiO_x_ (─P═O─Ni^2+^). The results demonstrate that PNPP still exhibits strong binding affinity toward PVK, with a binding energy of −0.549 eV (─N─O─Pb^2+^). We infer that PNPP forms strong chemisorption with both upper‐ and lower‐layer materials through its specific functional groups (Figure 2d), the N─O group of the nitro moiety acts as an electron donor, coordinating with Pb^2^⁺ ions in PbI_2_, while the P═O group of the phosphate moiety serves as a Lewis base site, forming a stable charge‐transfer complex with Ni^2^⁺ ions on the NiO_x_ surface.^[^ ^27^ ^]^ This dual‐site synergy not only exhibits significantly negative binding energies but, more importantly, establishes a continuous charge transport pathway at the interface, providing a solid theoretical basis for optimized energy level alignment. Molecular dynamics (MD) simulations further demonstrate pronounced aggregation of single SAM molecules on the NiO_x_ surface, as shown in Figure 2e, indicating poor interfacial compatibility and dispersibility. However, upon introduction of PNPP, its molecules effectively modify the NiO_x_ surface and markedly improve the dispersion of SAM (Figure 2f). This PNPP‐mediated interfacial modification strategy effectively suppresses the spontaneous aggregation of SAM molecules and promotes their uniform distribution on the NiO_x_ substrate.^[^ ^28^ ^]^

The influence of Co‐SAM on the crystallization behavior of perovskite was examined through scanning electron microscopy (SEM). As illustrated in Figure S2 (Supporting Information), Co‐SAM exerted minimal effect on the surface structure of NiO_x_ itself (Figure S11, Supporting Information). To investigate the morphology at the buried interface of the perovskite layer,^[^ ^29^ ^]^ the perovskite film was carefully peeled using ultraviolet (UV) glue (Figure S12, Supporting Information), allowing direct observation of the underlying surface (Figure 2e,f). The bottom surface of NiO_x_/Me‐4PACz revealed numerous small pinhole‐like defects concentrated at grain boundaries, with very few NiO_x_ particles detectable, suggesting a weak interfacial interaction between Me‐4PACz and the perovskite.^[^ ^30^ ^]^ In comparison, the NiO_x_/Me‐4PACz+PNPP interface displayed a markedly higher grain boundary density, with numerous fine NiO_x_ particles adhering to the perovskite, implying that PNPP molecules facilitated bonding, acting as a bridge to reinforce the connection between the NiO_x_ layer and the perovskite film.^[^ ^31^ ^]^ The bottom grain size also increased noticeably, from 428 nm in NiO_x_/Me‐4PACz to 507 nm with the inclusion of PNPP (Figure S11, Supporting Information). Further analysis of the perovskite top surface using SEM (Figure 2d–g) revealed that films deposited on NiO_x_/Me‐4PACz+PNPP exhibited a larger average grain size of 580 nm, whereas those formed on NiO_x_/Me‐4PACz had an average size of only 510 nm (Figure S13, Supporting Information) and presented more prominent inter‐grain voids.^[^ ^32^ ^]^ These findings suggest that Co‐SAM enhances crystal growth and suppresses defect formation during perovskite deposition. Cross‐sectional SEM images supported this observation (Figure 2h), revealing that perovskite films without Co‐SAM showed evident structural discontinuities. In contrast, the films formed on Co‐SAM‐modified substrates featured vertically aligned grains with reduced defect density, which is favorable for efficient charge transport across the film.^[^ ^33^ ^]^

**Figure 3:**
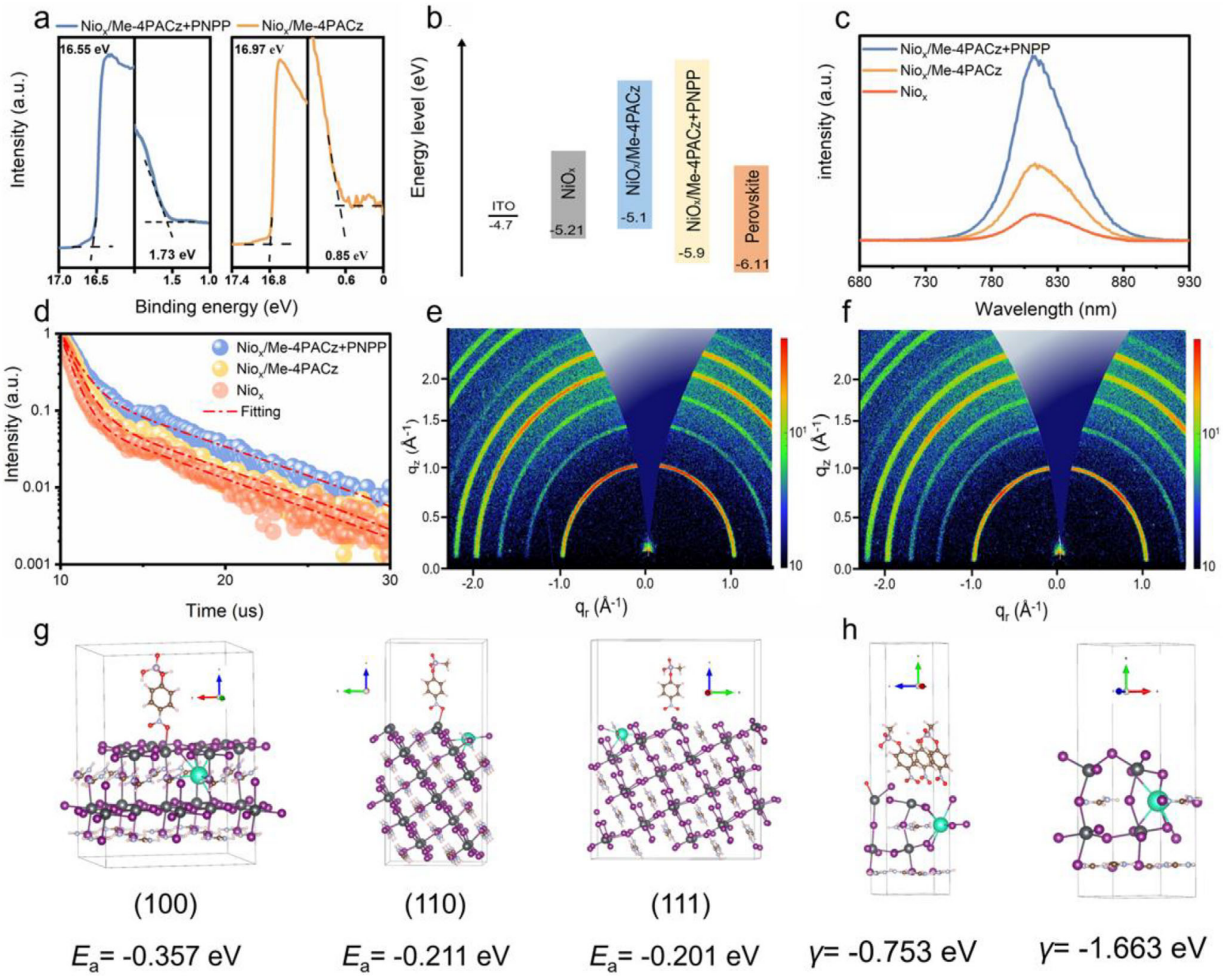
a) UPS spectra of the secondary electron cutoff (left) and the valence band (right) of ITO/NiO_x_/Me‐4PACz+PNPP and ITO/NiO_x_/Me‐4PACz substrates. b) Energy diagram for NiO_x_, NiO_x_/Me‐4PACz, and NiO_x_/Me‐4PACz+PNPP compared with perovskite. c,d) PL spectra, time‐resolved PL (TRPL) spectra of perovskite films deposited on NiO_x_, NiO_x_/Me‐4PACz, and NiO_x_/Me‐4PACz+PNPP. e,f) GIWAXS patterns of perovskite films deposited on NiO_x_/Me‐4PACz+PNPP and NiO_x_/Me‐4PACz. g) Calculated adsorption energy of the PNPP molecule with different crystal facets. h) Surface energy analysis with and without PNPP molecules. Where the pulper, red, dark pink, light pink, brown, silver, black, purple, and green atoms respectively represent the O, P, H, C, N, Pb, I, and Cs atoms.

Given that alterations in chemical states can markedly influence interfacial energy level alignment, UPS was employed to analyze the energy alignment between the ITO/Me‐4PACz and ITO/Me‐4PACz+PNPP systems relative to the perovskite light‐absorbing layer (**Figure** **3**a). The resulting energy level diagram for the ITO substrate, NiO_x_, Me‐4PACz,^[^ ^34^ ^]^ Me‐4PACz+PNPP, and perovskite is shown in Figure 3b. The data indicate that the work function of NiO_x_/Me‐4PACz+PNPP (−4.67 eV) is lower than that of NiO_x_/Me‐4PACz (−4.25 eV), and valence band maximum (VBM) of NiO_x_/Me‐4PACz+PNPP (−5.9 eV) lies deeper than that of NiO_x_/Me‐4PACz (−5.1 eV).^[^ ^35^ ^]^ This deeper energy alignment is beneficial for hole extraction, as the energy offset (ΔE) between the NiO_x_ layer and the perovskite valence band decreases significantly from 1.01 to 0.21 eV. In addition, the NiO_x_/Me‐4PACz +PNPP film demonstrates a substantially higher hole mobility of 4.224 × 10^−3^cm^2^ V*s* compared to 3.192 × 10^−3^ cm^2^ V*s* for the NiO_x_/Me‐4PACz film (Figure S14 and Table S2, Supporting Information). These findings indicate that the strong dual‐site coordination behavior of Co‐SAM at the NiO_x_/perovskite interface not only improves the electrical conductivity of the NiO_x_ layer but also fine‐tunes the energy level alignment while passivating undercoordinated Pb^2^⁺ surface defects.^[^ ^36^ ^]^ This enhanced interfacial contact facilitates more effective perovskite crystal nucleation and growth, thereby reducing non‐radiative recombination and significantly enhancing overall device performance.

To gain deeper insight into the impact of Me‐4PACz+PNPP on charge carrier behavior, steady‐state photoluminescence (PL) and time‐resolved photoluminescence (TRPL) tests were conducted. When comparing the perovskite film deposited on bare NiO_x_ to that modified with commercial Me‐4PACz, a notable enhancement in PL intensity was observed.^[^ ^37^ ^]^ This enhancement indicates that Me‐4PACz improves the interfacial energy level alignment and mitigates bulk defects, thereby reducing carrier recombination. Upon further modification with Me‐4PACz+PNPP, the PL intensity of the perovskite increased even more (Figure 3c), suggesting that the incorporation of PNPP molecules further refines energy level alignment, prolongs carrier lifetime, and effectively suppresses non‐radiative recombination.^[^ ^38^ ^]^ The TRPL spectra were fitted using a biexponential decay model (Figure 3d), and the average carrier lifetimes were extracted. The perovskite on bare NiOx exhibited an average lifetime of 131 ns. With Me‐4PACz treatment, this value rose to 201 ns and further extended to 395 ns with Me‐4PACz+PNPP incorporation (Table S3, Supporting Information). These findings confirm that the PNPP molecules not only retain the beneficial interfacial effects of SAM but also significantly lower interfacial defect density, thereby further reducing nonradiative losses and enhancing carrier dynamics at the interface.

The influence of Co‐SAM modification on the optical absorption behavior of perovskite films was assessed through ultraviolet–visible (UV–vis) absorption spectroscopy. As depicted in Figure S15 (Supporting Information), perovskite films deposited on NiO_x_ substrates treated with Me‐4PACz+PNPP and Me‐4PACz displayed enhanced absorption across the UV–vis spectrum. Notably, the absorption improvement was especially significant in the visible wavelength range of 500–600 nm, with Co‐SAM‐treated films exhibiting a more substantial enhancement.^[^ ^39^ ^]^ This observation was further validated by Tauc plot analysis (Figure S13, Supporting Information), confirming the improved optical absorption properties resulting from Co‐SAM modification. To evaluate the crystallographic quality of the perovskite films, grazing‐incidence wide‐angle x‐ray scattering (GIWAXS) measurements were performed (Figure 3e,f; Figure S17, Supporting Information). The GIWAXS patterns showed a characteristic scattering ring near *q* = 1.0 Å^−1^, indicative of randomly oriented α‐phase perovskite crystals. Films fabricated on Co‐SAM‐modified substrates exhibited significantly stronger (100) diffraction peak intensities compared to those treated with Me‐4PACz, suggesting enhanced crystal orientation and superior crystallinity induced by Co‐SAM.

These findings were further substantiated by XRD analysis (Figures S16 and S18, Supporting Information). Co‐SAM‐modified perovskite films displayed markedly intensified diffraction peaks relative to those modified with Me‐4PACz, confirming a substantial improvement in crystallinity.^[^ ^40^ ^]^ Additionally, a noticeable reduction in the intensity of PbI_2_‐related peaks was observed in the Co‐SAM‐modified films, indicating more efficient conversion of PbI_2_ into perovskite. The line profile analysis of the GIWAXS pattern revealed that in the Co‐SAM‐modified film, the characteristic peak of PbI_2_ at ≈q = 0.9 Å^−1^ was suppressed (Figure S19, Supporting Information), while a significantly enhanced diffraction intensity corresponding to the (100) lattice plane was observed. These findings indicate that the incorporation of the Co‐SAM promotes highly ordered and crystalline perovskite structures, improves the morphological quality and overall crystallinity of the film, and further confirms the beneficial role of Co‐SAM in optimizing the crystal quality of the perovskite layer.^[^ ^41^ ^]^

Based on the phenomenon of PNPP molecular triggering (100)‐oriented crystallization, we further analyzed the adsorption energy (E_a_) of PNPP molecules on different crystal planes, including (100), (110), and (111) facets. DFT calculation results demonstrate that PNPP exhibits the highest adsorption energy (−0.357 eV) on the (100) facet (Figure 3g), indicating a preferential adsorption of PNPP molecules on the (100) plane. The (100) facet possesses lower surface tension compared to the (110) and (111) facets, which is conducive to crystal nucleation/growth.^[^ ^42^ ^]^ Furthermore, the preferential adsorption of PNPP molecules can further reduce the surface energy (Figure 3h). Under the influence of this lowered surface energy, the (100) orientation dominates the crystallization process, ultimately leading to the formation of thermodynamically driven (100)‐oriented perovskite films.

**Figure 4:**
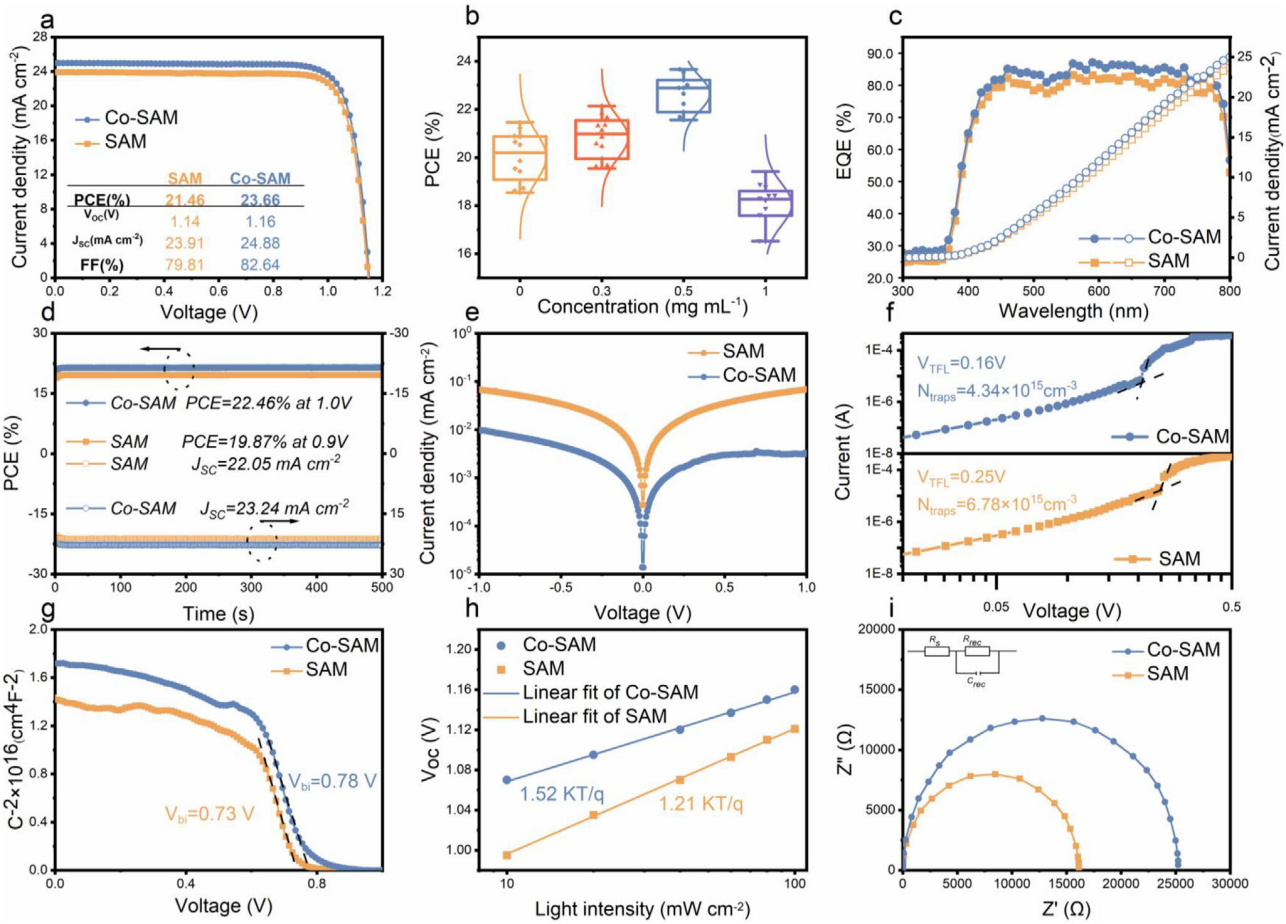
a) *J–V* curves of champion SAM and Co‐SAM F‐PSCs (reverse scan). b) PCE statistics of F‐PSCs with different amounts of PNPP treatment. c) EQE spectra. d) The steady‐state efficiency of devices. e) Dark *J–V* curves. f) SCLC‐voltage curve. g) Mott–Schottky plots. h) Light intensity dependence of *V*_OC_. i) Nyquist plots of the corresponding control and target devices, respectively.

To assess the impact of Co‐SAM modification on the performance of F‐PSCs, devices with a p‐i‐n configuration (PEN/ITO/NiO_x_/Me‐ 4PACz+PNPP/Perovskite/PC_61_BM/BCP/Au) were fabricated. The perovskite absorber layer had a thickness of ≈700 nm and a bandgap of ≈1.54 eV.^[^ ^43^ ^]^ **Figure** **4**a presents the current density–voltage (*J–V*) characteristics along with the corresponding performance parameters. The control device modified with conventional SAM achieved a PCE of 21.46%, with a *V*_OC_ of 1.14 V, a short‐circuit current density (*J*_SC_) of 23.91 mA cm^−2^, and a fill factor (FF) of 79.81%. Comparatively, the device incorporating Co‐SAM exhibited a substantially improved PCE of 23.66%, resulting from enhanced *J*_SC_ (24.88 mA cm^−2^), increased *V*_OC_ (1.16 V), and improved FF (82.64%). Comparative analysis with forward‐scan data (Figure S20, Supporting Information) reveals that the Co‐SAM treatment reduces the hysteresis index from 4.71% to 3.51%,^[^ ^44^ ^]^ primarily attributed to the dual functionality of PNPP passivation suppression at interfaces and enhanced charge carrier transport.^[^ ^45^ ^]^ To demonstrate the universality and reproducibility of this strategy, after applying Co‐SAM to small‐area (0.09 cm^2^) rigid devices, the efficiency increased from 23.55% to 24.80% compared to single SAM modification. Furthermore, through Co‐SAM modification, scalable large‐area rigid modules and flexible modules achieved efficiencies of 17.01% and 15.72% (Figure S21, Supporting Information), respectively, on an active area of 52.8 cm^2^, representing a significant improvement over the control group. Experimental data showed that the addition of small quantities of PNPP (0.3 or 0.5 mg mL^−1^) consistently improved the overall performance, likely due to more efficient charge extraction and suppression of nonradiative recombination,^[^ ^46^ ^]^ as summarized in Table S6 (Supporting Information). Statistical results of PCEs for devices treated with different PNPP concentrations are illustrated in Figure 4b, with average PCEs of 20.12%, 21.09%, 22.98%, and 18.19%, respectively. The distribution of *J–V* parameters for 15 devices under each condition is provided in Figure S22 (Supporting Information). Notably, increasing the PNPP concentration beyond 1 mg mL^−1^ did not result in further efficiency gains. Instead, excessive PNPP content adversely affected *J*_SC_, likely due to its insulating nature. However, even at higher concentrations, *V*_OC_ and FF remained superior to those of the control group, suggesting that Co‐SAM still improved energy level alignment and minimized recombination at the interface.^[^ ^47^ ^]^ Charge carrier lifetime and extraction capability were investigated via transient photovoltage (TPV) and transient photocurrent (TPC) measurements. Two types of devices were fabricated for this purpose. TPV measurements were carried out under open‐circuit conditions, and the results are presented in Figure S23a (Supporting Information). When the solar cell is exposed to pulsed light, photons generate electron‐hole pairs, leading to the production of a photovoltage. After the light pulse ceases, the voltage decays due to the recombination of electrons and holes at defect sites.^[^ ^48^ ^]^ The Co‐SAM modified device exhibited a longer decay time compared to the device modified with SAM alone, indicating that the passivation treatment effectively suppresses defect‐assisted non‐radiative recombination and prolongs the charge carrier life time. displays the short‐term photocurrent decay curves. In comparison to the SAM‐modified reference device, the Co‐SAM modified device shows a significantly shorter decay time (Figure S23b, Supporting Information), demonstrating that the passivation treatment not only suppresses recombination but also facilitates charge extraction and transport. This ensures that the device maintains a high FF and *J_SC_*, while simultaneously achieving an improved open‐circuit voltage.^[^ ^49^ ^]^ Figure 4c shows the external quantum efficiency (EQE) spectra, which validate the *J*_SC_ values derived from J–V measurements. The observed EQE enhancement throughout the visible range confirms improved charge carrier collection efficiency at the NiO_x_/perovskite interface. This improvement is attributed to better light absorption and effective passivation of buried interface defects facilitated by Co‐SAM. Stability tests at the maximum power point (MPP) revealed that the device retained a stable output of 22.46% over 500 s (Figure 4d), indicating strong operational stability. To understand the underlying mechanisms of performance enhancement with Co‐SAM, additional characterizations were conducted. Dark *J–V* curves revealed significantly lower leakage current in Co‐SAM‐modified devices (Figure 4e), indicative of reduced interfacial trap density and suppressed recombination. Space charge limited current (SCLC) analysis provided further insight: *V*_TFL_ dropped from 0.25 to 0.16 V, and the trap density (*N*_traps_) decreased from 6.78 × 10^15^ to 4.34 × 10^15^ cm^−3^ after Co‐SAM treatment (Figure 4f). Capacitance‐voltage (C‐V) measurements using Mott–Schottky analysis showed that the built‐in potential (*V*_bi_) increased from 0.73 to 0.78 V in the Co‐SAM‐modified device (Figure 4g),^[^ ^50^ ^]^ suggesting a stronger internal electric field that enhances charge separation and transport. Light‐intensity‐dependent *V*_OC_ analysis further demonstrated a lower slope (1.21 vs 1.52 *kT*/*q* for the control), confirming reduced trap‐assisted recombination (Figure 4h). Finally, electrochemical impedance spectroscopy (EIS) conducted under dark conditions (Figure 4i) indicated a higher recombination resistance (*R*_rec_) of 25.2 kΩ in the Co‐SAM device compared to 15.3 kΩ in the control, reinforcing the notion of suppressed carrier recombination.^[^ ^51^ ^]^ Collectively, these results establish that Co‐SAM enhances interface quality at the NiO_x_/perovskite junction, thereby facilitating efficient charge extraction and transport, which ultimately leads to notable improvements in F‐PSCs performance.

**Figure 5:**
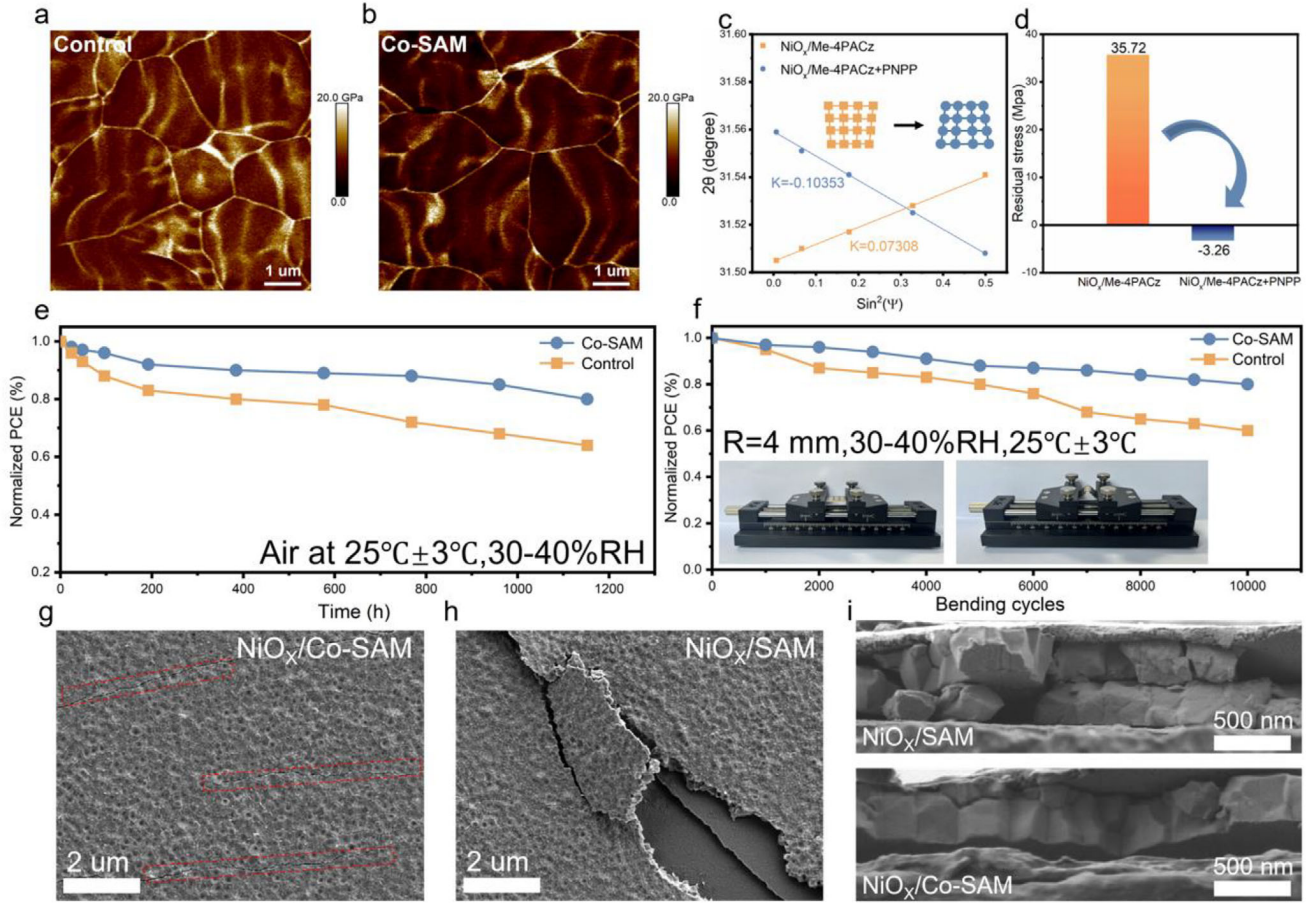
a,b) PFNMQ imaging of perovskite films on NiO_x_ substrates with and without PNPP‐modified Me‐4PACz layers. c) D‐spacing analysis indicating stress variation, with an inset illustrating the shift from tensile to compressive stress. d) Quantitative comparison of internal stress in differently modified perovskite films. e) Efficiency retention under ambient humidity over time, alongside diagrams of the flexible solar cell structure and its integration into a model aircraft. f) Performance stability during repeated bending at a 4 mm radius. g,i) Surface and cross‐sectional SEM images after 6000 bending cycles.

To further examine the mechanical characteristics of perovskite films, peak force quantitative nanomechanical mapping (PFQNM) imaging was conducted, as illustrated in **Figure** **5**a,b. Conventionally, solution‐processed perovskite films are characterized by a relatively high Young's modulus and limited mechanical flexibility, which makes them susceptible to cracking and delamination when subjected to bending stress.^[^ ^52^ ^]^ Experimental analysis revealed that Me‐4PACz and Co‐SAM films incorporating PNPP exhibited Young's moduli of 19.3 and 13.2 GPa, respectively. For the complete perovskite layers modified with Me‐4PACz and Co‐SAM, the measured Young's moduli were 9.2 and 4.3 GPa, respectively. These reductions in modulus indicate a marked improvement in film flexibility, confirming that Co‐SAM modification substantially enhances the mechanical robustness of F‐PSCs.^[^ ^53^ ^]^ This mechanical enhancement is primarily attributed to the structural features of PNPP molecules. The incorporation of a benzene ring between the carbazole moiety and the anchoring group increases molecular rigidity, which in turn strengthens intermolecular interactions. These stronger interactions promote the formation of denser, more crystalline films and help mitigate residual stress, thereby contributing to improved mechanical integrity and durability of the perovskite layer under mechanical deformation.

Given the notable influence of enhanced perovskite film morphology and crystallinity on residual stress, we analyzed the residual stress in the films using grazing‐incidence X‐ray diffraction (GIXRD) in conjunction with the 2*θ*–sin^2^ψ method, as shown in Figure S24 (Supporting Information). As the tilt angle ψ increased from 5° to 45°, the diffraction peaks of the control film gradually shifted toward lower 2*θ* values, while those of the Co‐SAM‐modified film exhibited a slight shift toward higher 2*θ* values.^[^ ^54^ ^]^ By evaluating GIXRD patterns across various tilt angles, a linear relationship between 2θ and sin^2^ψ was established, with the slope of the fitted curve serving as an indicator of residual stress (Figure 5c). The perovskite film deposited on the single SAM exhibited a positive slope in the 2θ–sin^2^ψ plot, reflecting the tensile stress. In contrast, the film modified with Co‐SAM showed a negative slope, indicative of compressive stress. Quantitatively, the residual tensile stress in the control film was calculated to be 35.72 MPa, whereas the Co‐SAM‐modified film exhibited a compressive stress of −3.26 MPa (Figure 5d). These findings confirm that the co‐assembly strategy not only improves film crystallinity but also effectively alleviates residual tensile stress, which in turn contributes to enhanced efficiency and mechanical stability of F‐PSCs.

To assess the humidity resistance of the devices, stability tests were conducted on unencapsulated samples under 35% relative humidity (RH), as illustrated in Figure 5e. After 1200 h of exposure, the Co‐SAM‐modified device maintained 81% of its original PCE, whereas the control device retained only 62%.^[^ ^55^ ^]^ Additionally, mechanical durability was evaluated through bending tests at room temperature (≈35% RH), as shown in Figure 5f. The operational stability of unencapsulated devices was evaluated under maximum power point (MPP) tracking and white light‐emitting diode (LED) illumination without UV filters. As shown in Figure S25 (Supporting Information), single‐SAM‐treated devices maintained 82% of their initial PCE over 300 h, while Co‐SAM‐modified devices exhibited significantly slower degradation, retaining >90% of initial performance after continuous 300‐h operation. At a bending radius (R) of 4 mm, the control device's PCE declined sharply with increasing cycles, retaining merely 59% of its initial efficiency after 10 000 cycles. Comparatively, the Co‐SAM‐modified device preserved 80% of its initial efficiency under the same conditions. Under simultaneous and sequential bending tests at radii of 2, 3, 5, and 6 mm (Figure S26, Supporting Information), the Co‐SAM modified devices consistently demonstrated superior performance retention, revealing remarkable mechanical stability across all curvature regimes, highlighting their superior mechanical robustness.^[^ ^27^ ^]^ When benchmarked against recently reported F‐PSCs in the literature (Table S7, Supporting Information), the devices developed in this study demonstrated comparable PCE and significantly enhanced long‐term operational stability, offering valuable strategies for the further development of high‐performance F‐PSCs. To corroborate these findings, scanning electron microscopy (SEM) characterization was conducted on devices subjected to 6000 bending cycles (Figure 5g–i). The SEM images revealed extensive cracking in the perovskite film on the Me‐4PACz‐modified substrate, while the Co‐SAM‐modified film exhibited only minor cracks^[^ ^56^ ^]^ and the results were consistent across multiple investigated areas (Figure S27, Supporting Information). Cross‐sectional SEM further confirmed the preservation of vertical grain orientation, indicating that Co‐SAM modification effectively improves mechanical integrity. Moreover, as presented in Figure S28 (Supporting Information), the UV–vis absorption intensity of PbI_2_/H_2_O solutions significantly decreased after PNPP addition, indicating a strong PbI_2_‐capturing capability. These results demonstrate that PNPP not only contributes to enhanced device performance through synergistic bottom interface engineering but also offers promising potential in lead recovery and environmental management applications, emphasizing its multifunctionality in perovskite solar cell systems.

Inspired by the outstanding performance of the devices, F‐PSCs were integrated into electronic devices and created a solar‐powered aircraft model (Figure S29, Supporting Information). This model can operate efficiently under outdoor sunlight and simulated solar conditions, powered by F‐PSCs.^[^ ^57^ ^]^ These results highlight the promising potential of flexible perovskite photovoltaic cells as mobile power sources, expanding their possible applications in a wide range of scenarios.

## 3 Conclusion

In summary, the introduction of PNPP into Me‐4PACz led to the development of an efficient Co‐SAM modification layer that optimizes the buried interface in F‐PSCs. The phosphate groups in PNPP effectively suppress surface defects on NiO_x_, while the nitro groups enhance wettability and passivate organic cation and halide vacancy defects at the bottom of the perovskite film. PNPP incorporation not only increases molecular rigidity but also promotes the formation of denser films through strengthened intermolecular interactions, improving perovskite crystallinity and alleviating residual stress. The Co‐SAM modification improves film morphology and compositional homogeneity, mitigating tensile stress and enhancing mechanical reliability. As a result, this study successfully optimizes interface engineering and enhances the performance of F‐PSCs through the Co‐SAM strategy. The F‐PSCs incorporating Co‐SAM afforded a champion PCE of 23.66% and scalable large‐area rigid modules and flexible modules achieved efficiencies of 17.01% and 15.72%. This approach significantly improves energy level alignment, defect passivation, and charge carrier transport at the NiO_x_/perovskite interface. Experimental results show that the Co‐SAM modification not only enhances perovskite film crystallinity but also substantially improves device stability by releasing residual stress and increasing mechanical flexibility.

## Conflict of Interest

The authors declare no conflict of interest.

## Supporting information



Supporting Information

## Data Availability

The data that support the findings of this study are available from the corresponding author upon reasonable request.
