# Supplementary material for: Co‐Self‐Assembled Interface Engineering Assisted for Bend‐Resistant and Efficient Flexible Perovskite Solar Cells
Source: Adv Sci (Weinh). 2025 Oct 24;13(3):e09724. doi: 10.1002/advs.202509724 (PMC12806289; doi:10.1002/advs.202509724)
Supplement: Supplementary file 1 — Supporting Information [file ADVS-13-e09724-s001.docx]

**Supporting Information**

**Co-self-assembled Interface Engineering Assisted for Bend-resistant and Efficient Flexible Perovskite Solar Cells**

Chunlong Wang, Chu Zhang ^*^, Qingxue Wang, Hao Li, Yutong Wu, Yue Zhao, Shennan Chen, Liang Li, Mingjun Nie, Jiaxing Song, Zaifang Li, Yonggang Yu, Lei Shi*, Yongchun Ye, Yu Wang, Tingli Ma, Wensheng Yan^*^

**Materials**

Isopropyl alcohol (99.5%, Superdry)，Ethanol(99.5%, Superdry) were purchased from Shanghai J&K Scientific Co. Dimethyl sulfoxide (DMSO，99.9%) and N，N-dimethylformamide (DMF, 99.8%) were purchased from Alfa Aesar and Sigma - Aldrich respectively. PEN substrates (transmission greater than 95%), Chlorobenzene (99.8%) and PbI_2_ (99.999%) were purchased from Advanced Election Technology Co., Ltd. Formamidinium iodide (FAI，99.98%) were purchased from Greatcell Solar Materials. Cesium iodide (CsI，99.9%) were purchased from Advanced Election Technology CO.，Ltd.Nickel oxide (NiO_x_) nanoparticle was purchased from XI’AN E-Light New Material CO.，Ltd. PC_61_BM were purchased from Nano-C in USA，Phenethylamine iodide (PEAI) and Bathocuproine (BCP) were purchased from Xi’an Polymer Light Technology in China. phosphonic acid mono-(4-nitrophenol) ester (PNPP >98.0%) were purchased from Aladdin CO.，Ltd. [4- (3,6-Dimethyl-9H-carbazol-9-yl) butyl] phosphonic Acid (Me-4PACz >99.0%) were purchased from TCI Chemicals CO.，Ltd.

**Fabrication of small-area devices:**

The PEN substrates was cleaned by sequentially sonicating for 15 minutes each using ethanol, deionized water, acetone and ethanol. hen, use fire-resistant double-sided adhesive to attach PEN substrates onto the rigid glass. Add 20 mg of NiO_x_ nanoparticles to 1 mL of deionized water, preparing a 20 mg/ml NiO_x_ NPs aqueous ink. Add 40% mol H_2_O_2_ into the ink to improve the dispersion of NiO_x_ NPs and prevent particle aggregation. Subsequently, sonicate the prepared ink for 10 minutes. First, pass the ink through a 0.22 μm pore-sized PTFE (hydrophilic) filter. Subsequently, apply the filtered ink to PEN substrates using a spin coater at 4000 rpm for 30 seconds. Next, anneal the NiO_x_ films at 100 °C for 10 minutes in ambient conditions. Immediately after annealing, transfer the NiO_x_ films into a nitrogen-filled glove box. The self-assembled monolayers (SAM) or Co-SAM were fabricated by spin-coating the Me-4PACz (0.5 mg/mL in ethyl alcohol) and phosphonic acid mono-(4-nitrophenol) ester (PNPP, 0-1mg/mL) on NiO_x_ substrates at 4000 rpm for 30 s, followed by thermal annealing at 100℃ for 10 min. After that, the substrates needed to be washed with ethanol through spin coating at 4000 rpm for 30 s, then annealing at 100℃ for 5 min. To create films of FA0.95Cs0.05PbI3 perovskite, a 1.4 M precursor solution was prepared by dissolving 228.4 mg of FAI, 18.2 mg of CsI, and 645.4 mg of PbI_2_, in a mixed solvent of DMF and DMSO at a 4:1 volume ratio. This precursor solution was first spin-coated onto a substrate at 2000 rpm for 10 seconds, followed by 4000 rpm for 40 seconds. During the latter stage, 150 µL of CB was added to the film 5 seconds before the spin-coating process ended. The resulting wet films were then annealed at 100°C for 30 minutes. A solution of PEAI in isopropanol was prepared at a concentration of 1 mg/ml and spin-coated onto the perovskite film at 5000 rpm for 20 seconds, followed by annealing at 100℃ for 10 minutes. Next, a PC_61_BM solution in CB at a concentration of 23 mg/mL was spin-coated at 2500 rpm for 40 seconds. This was followed by spin-coating a BCP solution in IPA at 5 mg/mL on the PC_61_BM layer at 5000 rpm for 30 seconds. In the vacuum chamber, a 120 nm thick Au electrode was thermally evaporated through a metal shadow mask with a 0.09 cm² square aperture (see the design in Figure S30). Finally, the flexible inverted devices were peeled off from the glass substrate. The active area of these devices is 0.09 cm².

**Fabrication of flexible solar modules:**

The flexible solar module comprising 11 subcells was fabricated using P1, P2, and P3 laser scribing lines with a wavelength of 1064 nm. The pre-patterned PET/ITO substrates were pre-scribed with P1 lines (approximately 200 μm wide), followed by cleaning with acetone and isopropanol to remove adhesive residues. A NiO_x_ solution was blade-coated onto the ITO substrate at a speed of 10 mm/s to form the NiO_x_ HTL, which was then annealed at 100 °C for 10 minutes. Subsequently, SAM and Co-SAM layers were sequentially blade-coated onto the NiO_x_ substrate at a speed of 10 mm/s. For perovskite deposition, the precursor solution was dispensed ahead of the blade and coated at 20 mm/s. The wet film was immediately treated with flash evaporation for 38 seconds to accelerate solvent removal. The perovskite film was then transferred to a hotplate and annealed at 100 °C for 15 minutes. A C_60_ layer (25 nm) and a BCP interlayer (8 nm) were sequentially thermally evaporated in a high-vacuum chamber (<4×10^-4^ Pa). P2 and P3 scribing lines were patterned using a 355 nm picosecond laser system. The P2 lines (approximately 100 μm wide) were achieved with an average laser power of 12 W and a pulse repetition frequency of 65 kHz. After electrode deposition, the P3 lines (approximately 80 μm wide) were patterned under an average laser power of 0.78 W and a pulse repetition frequency of 80 kHz. The spacing between P1, P2, and P3 lines was maintained at approximately 30 μm. The active area was determined to be 52.8 cm² by calculating the difference between the shadow mask area and the dead zone.

**Measurement and characterization**

Calculate the binding energy, HOMO, and LUMO energy levels of the PNPP molecule using GaussView and Gaussian09W. The *J-V* curve of PSCs was measured by a standard xenon solar simulator (Peccel Tech., Inc., Japan) with standard solar illumination (AM 1.5G, 100 mW cm^−2^). The source table uses the Keithley 2601 B light source (TEKTRONIX, INC., USA) The Monochromatic incident photon-to-electron conversion efficiency (IPCE) was measured by PEC-S20 of Peccel Tech., Inc. X-ray photoelectron spectroscopy (XPS) was conducted on a Thermo ScientificTM K-AlphaTM+ spectrometer equipped with a monochromatic Al Kα Xray source (1486.6 eV) operating at 100 W. Ultra-violet photoelectron spectroscopy (UPS) measurements were conducted with the same equipment employing He I (21.22 eV) excitation. For measuring the secondary electron cutoff, a 5 V bias was applied to the substrate. The transient-state photoluminescence (PL) and time-resolved photoluminescence (TRPL) were recorded by transient fluorescence spectrometer FLS980, Edinburgh Instruments. The UV-Vis absorption/transmittance properties were measured by a Lambda 750S UV spectrophotometer (PerkinElmer, UK) with a tungsten lamp as light source. The Electrochemical Impedance Spectroscopy (EIS) and Mott-Schottky curve characterizations were performed on a CHI600E electrochemical workstation. The FTIR spectrum was acquired with a Thermo Scientific Nicolet iS20 in the range of 4000–500 cm^-1^ . The microscopic morphology of the as-prepared perovskite films was taken with a high-resolution scanning field electron microscope (SEM, Hitachi SU70). The Atomic force microscopy (AFM) images were performed by Bruker Dime. Grazing incidence wide-angle scattering (GIWAXS) at an incidence angle of 0.4° was performed at BL17B1 beamline of Shanghai Synchrotron Radiation Facility (SSRF) using the X-ray energy of 10 KeV. Raman spectroscopy measurements were performed on a Horiba LabRAM HR Evolution system (Japan). Solution-state nuclear magnetic resonance (NMR) spectroscopy was conducted on a Bruker Avance III 400 MHz spectrometer (Germany). The surface photovoltage spectrum was measured using a CEL-SPS1000 surface photovoltage testing system. For the measurements, non-equilibrium carriers in the cell are excited by 660 nm (CNI-laser, 1 kHz, 20 ns) pulse laser. A digital oscilloscope (Tektronix, DPO 7104) is used to record the photocurrent decay process with a sampling resistor of 50 Ω.

Stability test: The complete PSCs could be encapsulated in a N_2_ glove box. The operational stability of the encapsulated cells was measured at 55 ℃ under a white light emitting diode lamp with sixteen-channel thin 4 film photovoltaic maximum power point tracking test system (YH-VMPP-16).

**DFT calculations:**

First principles theory calculations are carried out using the Vienna Ab initio Simulation Package (VASP) within the framework of the generalized gradient approximation functional. Projected augmented wave (PVW) potentials are used to describe the ionic cores and take valence electrons into account using a plane wave basis set with a kinetic energy cutoff of 400 eV. K-points are sampled under the Monkhorst-Pack scheme for the Brillouin-zone integration (K-points were sampled using the Gamma Point). In all calculations, the forces acting on all atoms are < 0.02 eV/Å in fully relaxed structures, and self-consistency accuracy of 10^-5^ eV is reached for electronic loops. The surface is modeled as a periodic slab consisting of at least five atomic layers separated by a vacuum of at least 25 Å in the direction normal to the surface.

The electrostatic potential of the SO was calculated using the Gaussian 16 program (Revision C.01) with the B3LYP functional^[14]^. All-electron 6-311+G(d,p) basis sets were applied for C, H, O, and N. Additionally, the Grimme-D3 scheme was employed to account for Van der Waals interactions.

**Mott-Schottky analysis:**

The capacitance-potential relationship obeys the Mott-Schottky Equation.

$$\frac{1}{C^{2}}=\frac{2}{e\varepsilon\varepsilon_{0}}(V_{bi}-V-\frac{K_{B}T}{e})$$

where C refers to the capacitance of the space charge region, V_bi_ represents the built-in potential, V is the applied potential, kB is the Boltzmann constant, T is the thermodynamic temperature, and ND is the donor density. The built-in potential can thus be estimated from the intercept of a Mott-Schottky plot with the x-axis.

***V*_oc_ versus light intensity curves calculation**

the ***V*_oc_** ought to possess a liner correlation with the logarithm of light intensity according to Equation

$$\frac{\left( dV_{OC} \right)}{\mathrm{dlgI}}=2.303(nk_{B}T)/q$$

where k_B_ is the Boltzmann constant, n is the ideality factor, T is the thermodynamic temperature, and q is the elementary charge.

As shown in Tables S1, the conductivity of the PNPP-modified Me-4PACz film increased from 4.34×10^-3^ to 5.72×10^-3^. The hole mobility was obtained through the following equation transformation:

$J=9\varepsilon_{0}\varepsilon_{r}\mu V^{2}L^{3}$/8

The hole mobility increased from 3.192×10^-3^ to 4.224×10^-3^ cm^2^V^-1^s^-1^ The modification with PNPP not only had no negative impact on the conductivity and hole mobility of the film but actually improved them, indicating enhanced hole extraction and transport efficiency. Specific data on conductivity and hole mobility can be found in Tables S1 and S2.

**Residual tensile force：**

Change the tilt angle (Ψ = 0°, 15°, 25°, 35°, 45°), get the X-ray diffraction images of perovskite at different depths, and get the relationship between the interplanar distance dn and Sin2Ψ. The release of residual stress in the perovskite film is estimated by the slope of the dn-Sin2Ψ fitting curve:

𝜎𝑅 = (𝐸1+𝑣)(𝑚𝑑𝑛)

where σR is the residual stress of the film; E is the Young's modulus of the perovskite

film ; v is the Poisson's ratio ; m is the slope of the straight line after linear fitting; dn is the intercept of the straight line after linear fitting.

**EQE analysis:**

It has been shown that the EQE of the EL of the solar cells under bias voltage

(operating as a light-emitting diode (LED)) can be used to estimate the recombination in the devices. Higher radiative recombination emission efficiency could deliver high *V*_OC_ based on the equation:

𝑉_𝑂𝐶_ = (𝑛𝑘_𝐵_𝑇/𝑞) 𝑙𝑛 [𝐸𝑄𝐸_𝐸𝐿_ (𝐽_𝑝ℎ_/𝐽_𝑒𝑚,0_) + 1]

where EQEEL is quantum efficiency when the injection current in the dark is equal to the photocurrent (Jph) of the device under light illumination and Jem,0 is the current of re-emitted photons at room temperature.

**Table S1.** Calculated conductivity parameters of the Me-4PACz film before and after modification with PNPP.

| ETL/SCLC | Area（cm^-2^） | Slop（I/V） | Conductivity  （mS cm^-1^） |
| --- | --- | --- | --- |
| ITO/NiO_x_/Me-4PACz(Control) | 0.09 | 0.19260 | 4.342×10^-3^ |
| ITO/NiO_x_/Me-4PACz+PNPP(Target) | 0.09 | 0.23008 | 5.724×10^-3^ |

**Table S2.** Calculated hole mobility parameters of the Me-4PACz film before and after modification with PNPP.

| ETL/SCLC | Ε | Slop（I/V） | Mobility  (cm^-2^V^-1^S^-1^) |
| --- | --- | --- | --- |
| ITO/NiOx/Me-4PACz(Control) | 14 | 0.25732 | 3.192×10^-3^ |
| ITO/NiO_x_/Me-4PACz/PNPP(Target) | 14 | 0.21937 | 4.224×10^-3^ |

**Table S3.** Summary of the PL lifetime parameters from fitting curves of the TRPL decay measurements

| **Samples** | ***A_1_*** | ***τ_1_/***  ***ns*** | ***A_2_*** | ***τ_2_/***  ***ns*** | ***Weighted average τ /***  ***ns*** |
| --- | --- | --- | --- | --- | --- |
| NiO*_x_* / Perovskite | 0.58865 | 78.42321 | 0.41135 | 213.23543 | 131 |
| NiO*_x_* /Me-4PACz/ Perovskite | 0.48253 | 64.95034 | 0.51747 | 341.39903 | 201 |
| NiO*_x_* / Me-4PACz+PNPP/Perovskite | 0.31947 | 123.32126 | 0.68053 | 523.46324 | 395 |

Notes: The time-resolved PL decay curves measured by time-correlated single-photon counting were fitted by a bi-exponential equation: Y=*A*_1_exp(−*t*/*t*_1_)+*A*_2_exp(−*t*/*t*_2_). Here, *A*_1_ and *A*_2_ correspond to the decay amplitudes of fast and slow components, respectively, where *A*_1_+*A*_2_=1. The average lifetime *t*_avg_ was calculated by *t*_avg_=(*A*_1_*t*_1_+*A*_2_t_2_)/(*A*_1_+*A*_2_).

**Table S4.** Summary of relative composition of various nickel species from peak fitting of the nickel 2*p*3/2 core level spectra.

| Sample | Ni species | *Peak area* | *B. E / eV* | *Area ratio* |
| --- | --- | --- | --- | --- |
| NiO_x_/Me-4PACz+PNPP | Ni^2+^ | 57125.23 | 853.078 | 0.34 |
|  | Ni^3+^ | 164193.12 | 855.65 | 0.99 |
| NiO_x_/Me-4PACz | Ni^2+^ | 55557.19 | 854 | 0.28 |
|  | Ni^3+^ | 194984.88 | 855.78 | 1.00 |

**Table S5.** Summary of relative composition of various oxygen species from peak fitting of the elemental lead 4f core level spectra.

| Sample | Pb species | *Peak area* | *B. E / eV* | *Area ratio* |
| --- | --- | --- | --- | --- |
| **PbI_­2_** | Pb 4f_5/2_ | 201998.05 | 143.22 | 0.82 |
|  | Pb 4f_7/2_ | 246511.83 | 138.35 | 1 |
|  | Pb^0­^ | 66813.80 | 141.52 | 0.27 |
|  | Pb^0^ | 83379.37 | 136.64 | 0.34 |
| **PbI_2_+PNPP** | Pb 4f_5/2_ | 201198.51 | 142.67 | 0.81 |
|  | Pb 4f_7/2_ | 247686.28 | 137.81 | 1 |
|  | Pb^0^ | 34454.77 | 140.82 | 0.14 |
|  | Pb^0^ | 61178.15 | 136.05 | 0.25 |

**Table S6.** Experimental data graphs for different concentrations of PNPP.

| Concentration of PNPP | *V_oc_* / V | *J_sc_* / mA/cm^2^ | *FF / %* | *PCE* / % |
| --- | --- | --- | --- | --- |
| 0 mg/mL | 1.14 | 23.91 | 79.81 | 21.46 |
| 0.3 mg/mL | 1.14 | 24.22 | 80.66 | 22.27 |
| 0.5 mg/mL | 1.16 | 24.88 | 82.64 | 23.66 |
| 0.7 mg/mL | 1.13 | 22.89 | 80.12 | 20.72 |
| 1 mg/mL | 1.11 | 20.84 | 79.52 | 18.39 |

**Table S7.** Statistics of some parameters and processes involved in Ref.1-6

| Device  Structure | Year | PCE | Stability | Ref. |
| --- | --- | --- | --- | --- |
| PEN/ITO /SnO2/FDCA/perovskite/PEAI/Spiro-OMeTAD/Ag^[1]^ | 2024 | 24.53%  0.09 cm^2^ | RH=40%,500h, 90%  r=5mm,10000r, 81% | 1 |
| PEN/ITO /SnO2/ BPySCN /perovskite/PEAI/Spiro-OMeTAD/Au^[2]^ | 2024 | 23.70%  0.09 cm^2^ | RH=40%,600h, 89.6%  r=4mm,5000r, 89.7% | 2 |
| PEN/ITO/MeO-2PACz TTP/perovskite/C60/BCP /Au^[3]^ | 2024 | 23.81%  0.04 cm^2^ | RH=15%,1050h, 96.2%  r=4mm,2500r, 92% | 3 |
| PEN/ITO/SnO2/DNS/Perovskite/Spiro-OMeTAD/Au^[4]^ | 2024 | 24.47%  0.09 cm^2^ | RH=30%,3000h, 91%  r=4mm,4000r, 90% | 4 |
| PEN/ITO/SnO2/ perovskite/ADP/Interfacial layer/Spiro-OMeTAD/Ag^[5]^ | 2024 | 23.53%  0.09 cm^2^ | RH=35%,1300h, 91%  r=5mm,8000r, 90% | 5 |
| PEN/ITO/SnO2/perovskite/interfacial layer/spiro-OMeTAD/Ag^[6]^ | 2024 | 24.04%  0.081 cm^2^ | RH=30%,1200h, 90.6%  r=2mm,10000r, 90% | 6 |


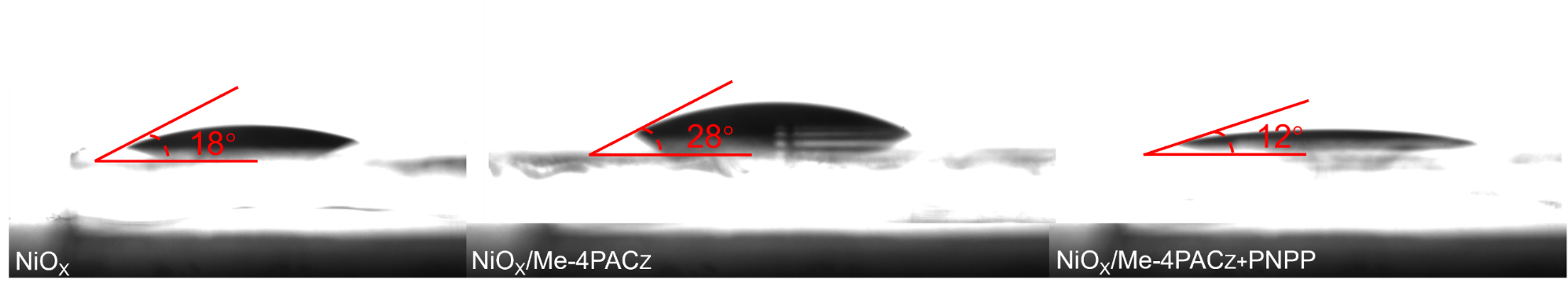


F**igure S1.** Contact angles measured with perovskite precursor on NiO_x_, NiO_x_/Me-4PACz, and NiO_x_/Me-4PACz+PNPP.


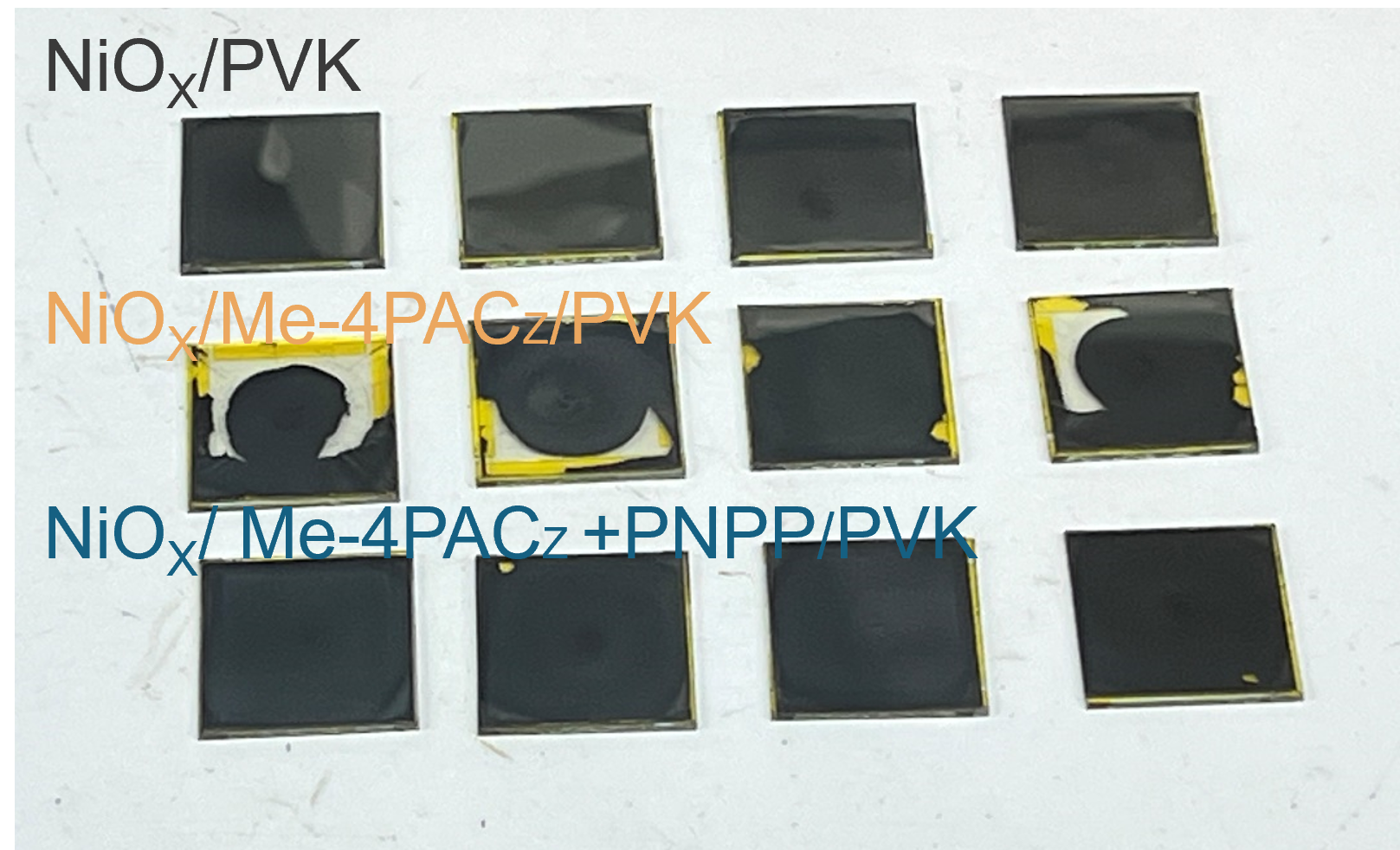


**Figure S2.** The spin-coating conditions of perovskite films on substrates NiO_x_, NiO_x_/Me-4PACz, and NiO_x_/Me-4PACz+PNPP.


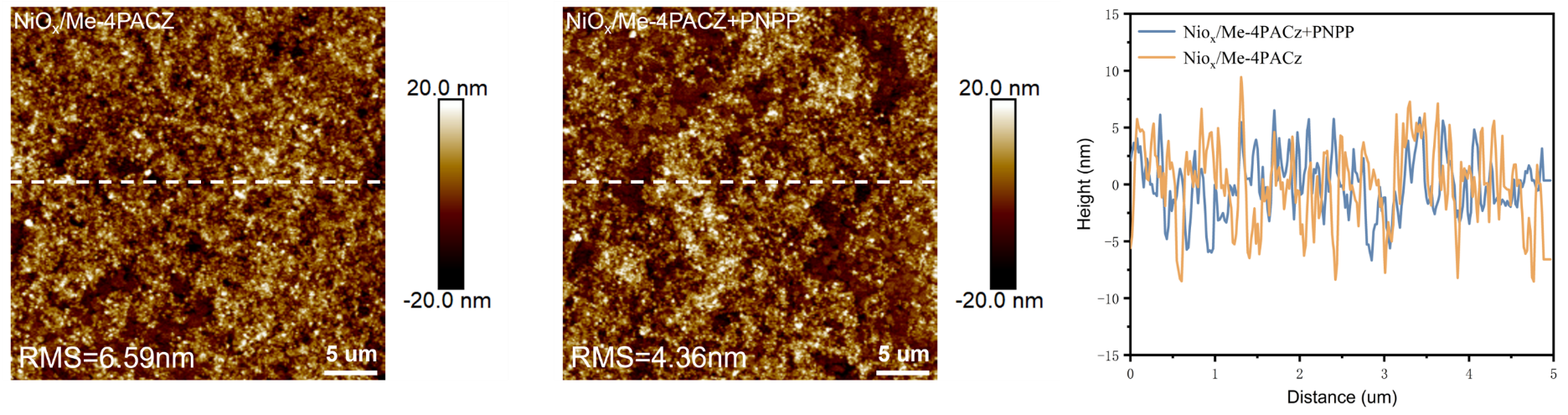


**Figure S3.** AFM morphology images of NiO_x_/Me-4PACz and NiO_x_/Me-4PACz+PNPP. The high and low distribution trend chart corresponds to the white line in AFM.


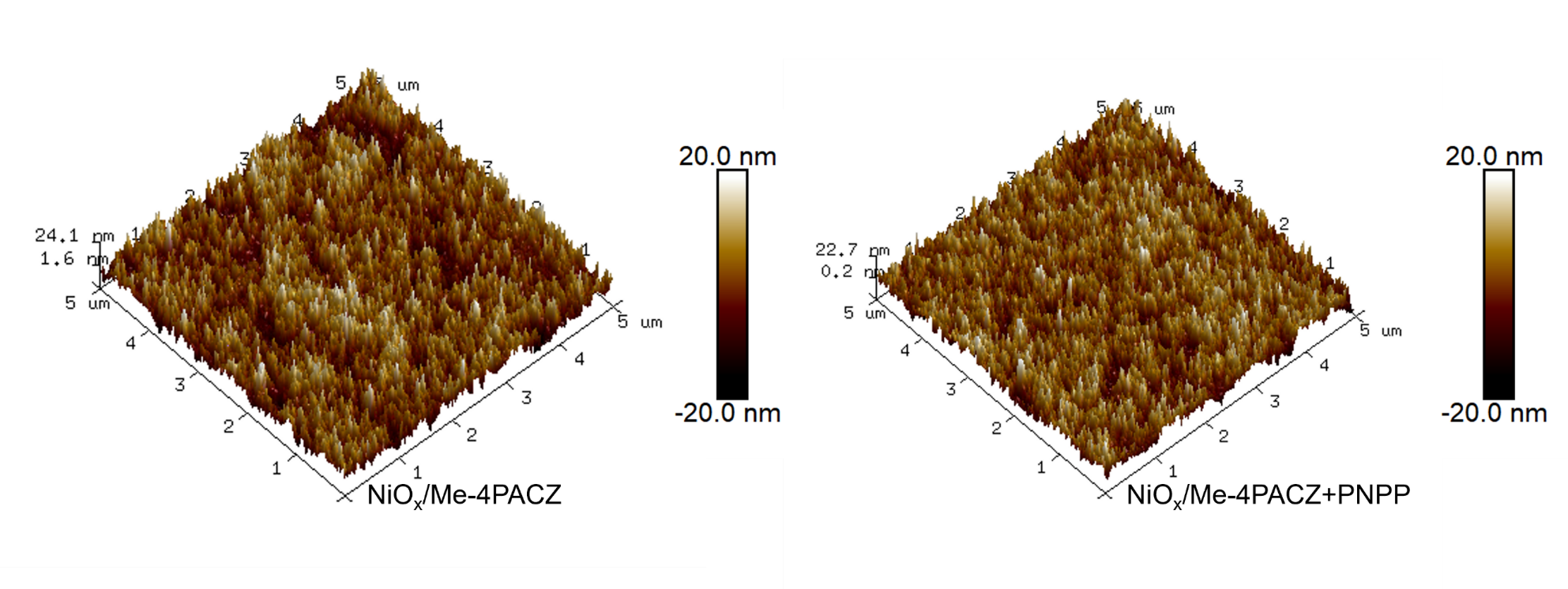


**Figure S4.** The 2D AFM morphology images of NiO_x_/Me-4PACz and NiO_x_/Me4PACz+PNPP.


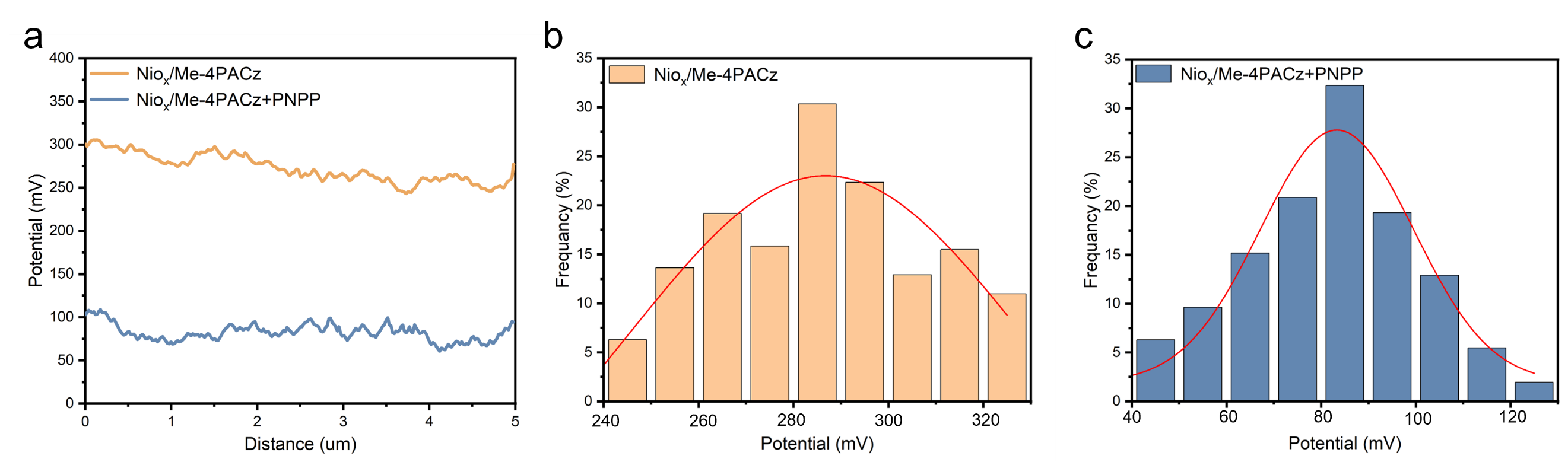


**Figure S5.** Mapping of KPFM on the surface of NiO_x_/Me-4PACz+PNPP, NiO_x_/Me-4PACz. and corresponding the surface potential distribution analysis.

**
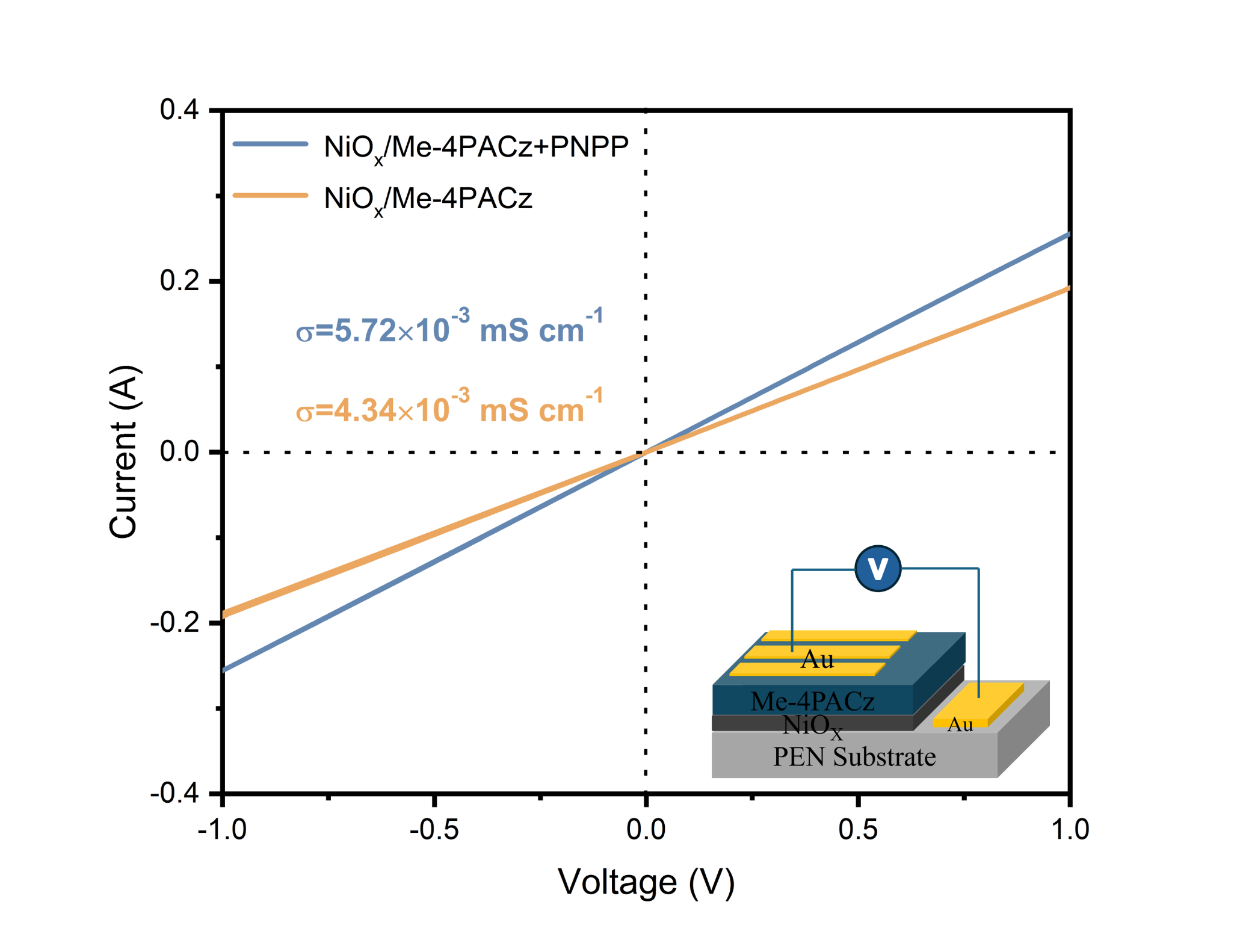
**

**Figure S6.** Electrical conductivity of the NiO_x_/Me-4PACz+PNPP and NiO_x_/Me-4PACz.


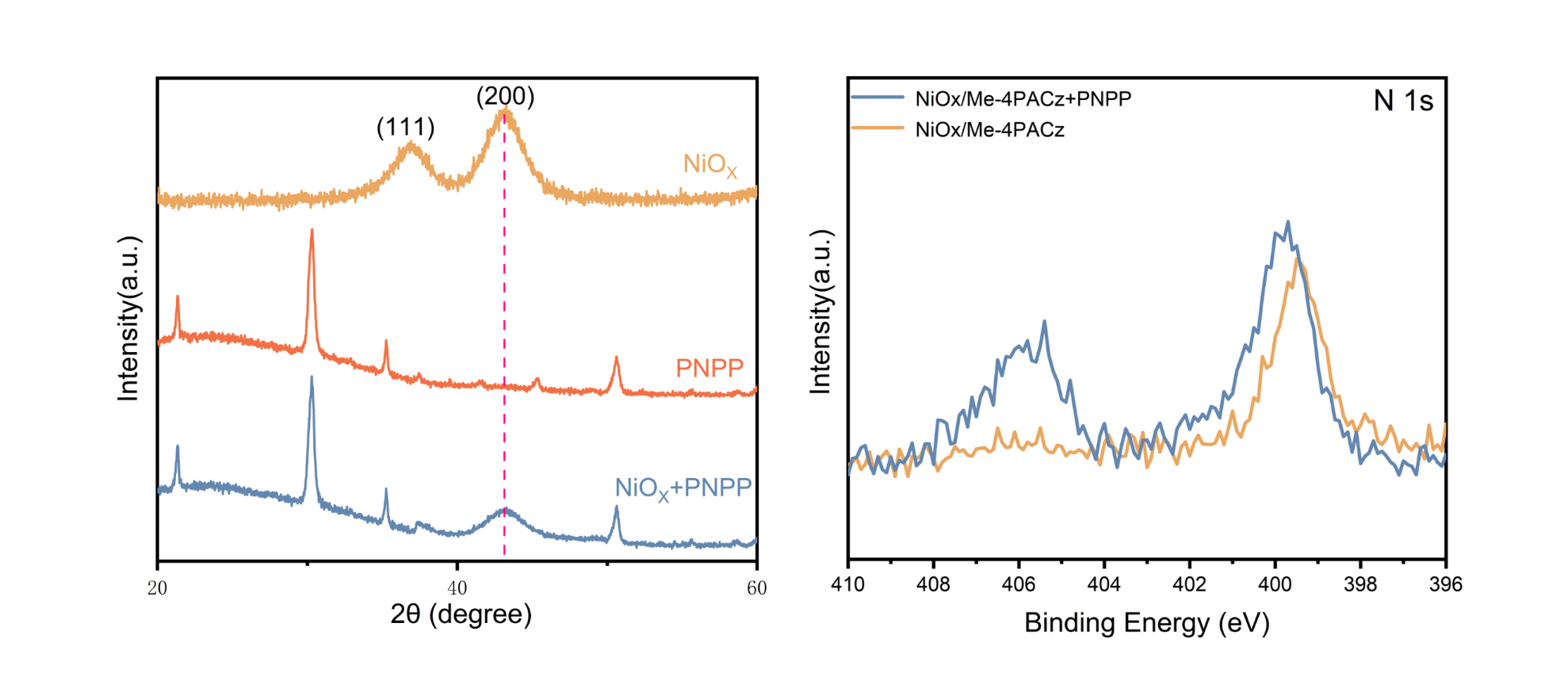


**Figure S7.** XRD of NiO_x_, PNPP, and NiO_x_+PNPP. N 1s for NiO_x_/Me-4PACz, NiO_x_/Me-4PACz+PNPP.


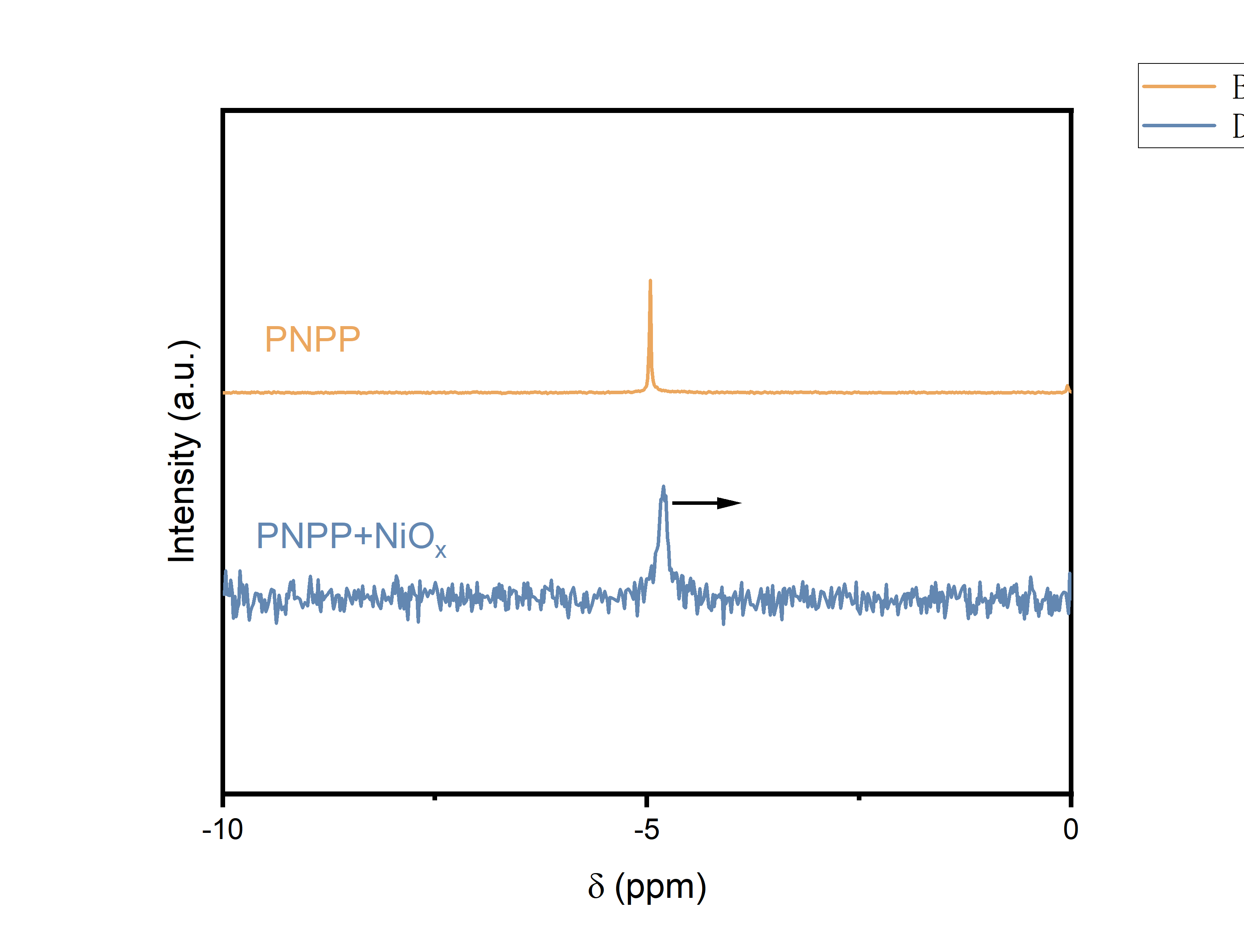


**Figure S8.** ^31^P-NMR spectra of neat PNPP and PNPP-NiO_x_ mixed solutions.


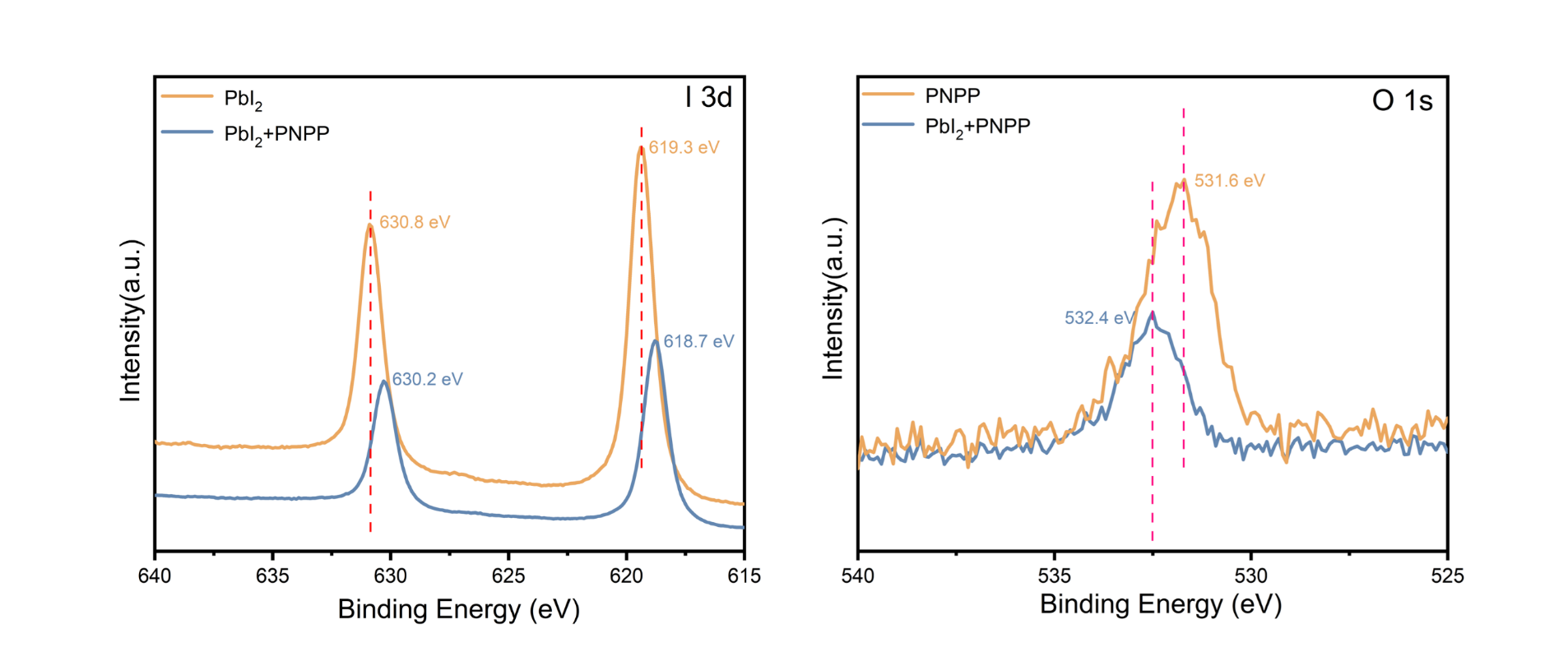


**Figure S9.** XPS of I 3d and O 1s for PNPP+PbI_2_ and PbI_2_.


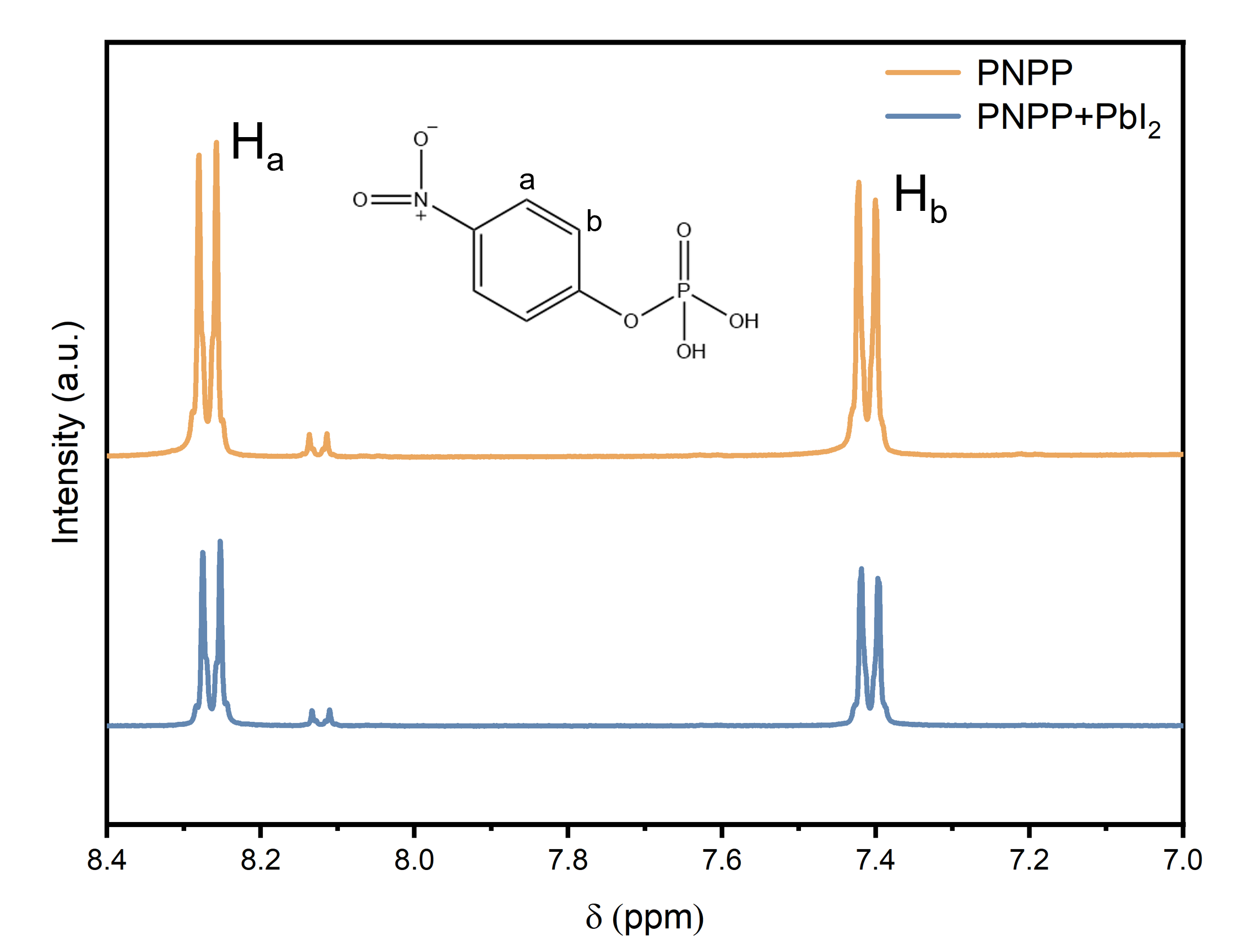


**Figure S10.** ^1^H-NMR spectra of neat PNPP and PNPP-PbI_2_ mixed solutions.


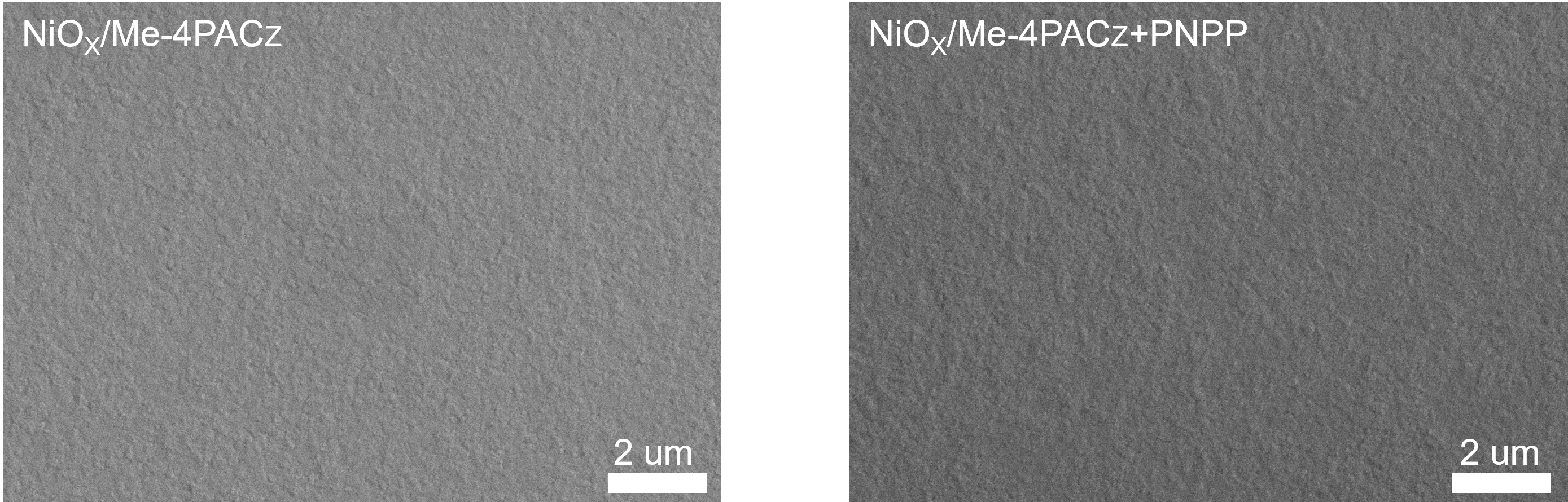


**Figure S11.** Top-view SEM images of the NiO_x_/Me-4PACz and NiO_x_/Me-4PACz+PNPP.


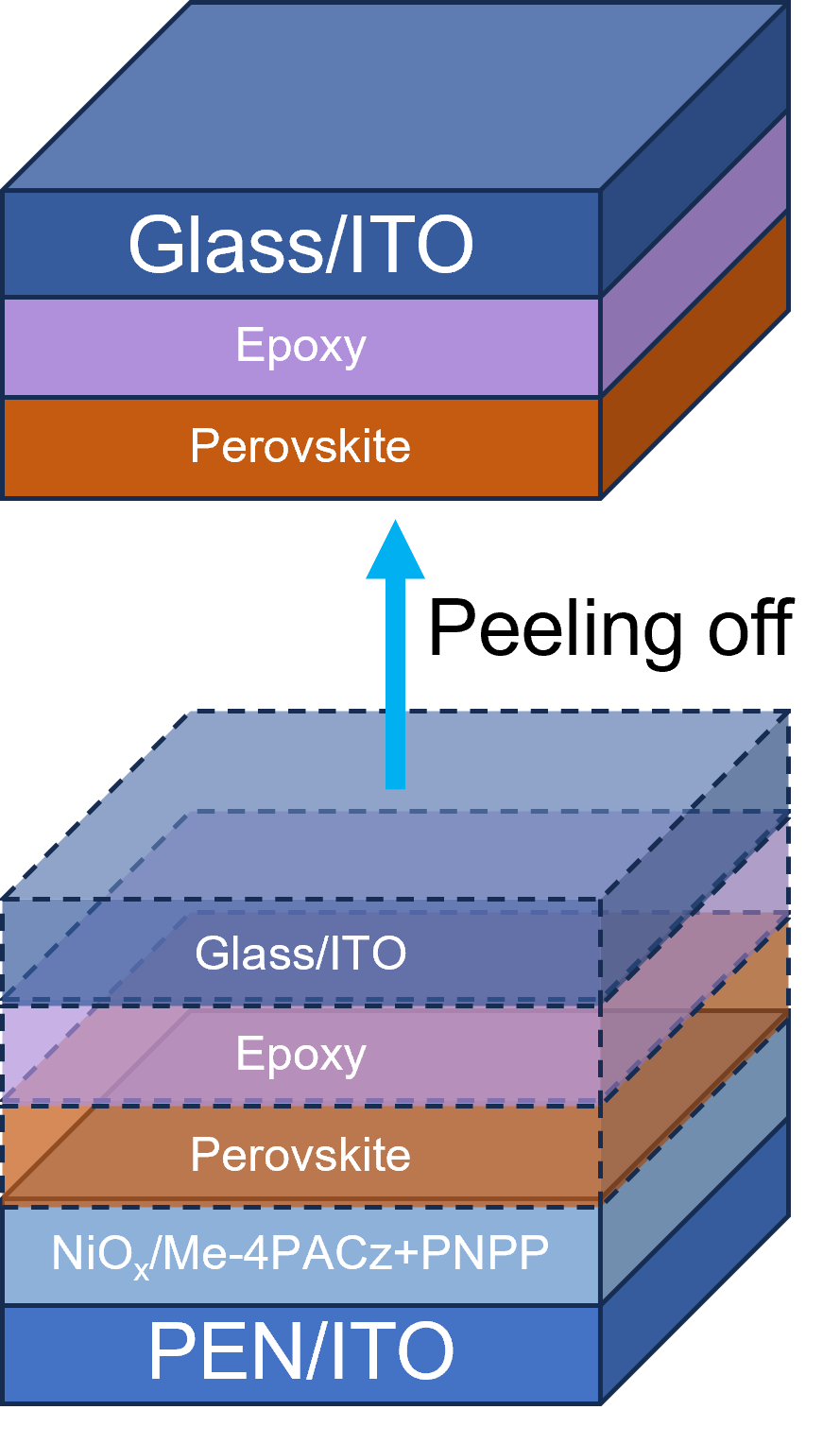


**Figure S12**. Schematic illustration of peeling the perovskite film from PEN/ITO substrates with an epoxy encapsulant for SEM characterization.


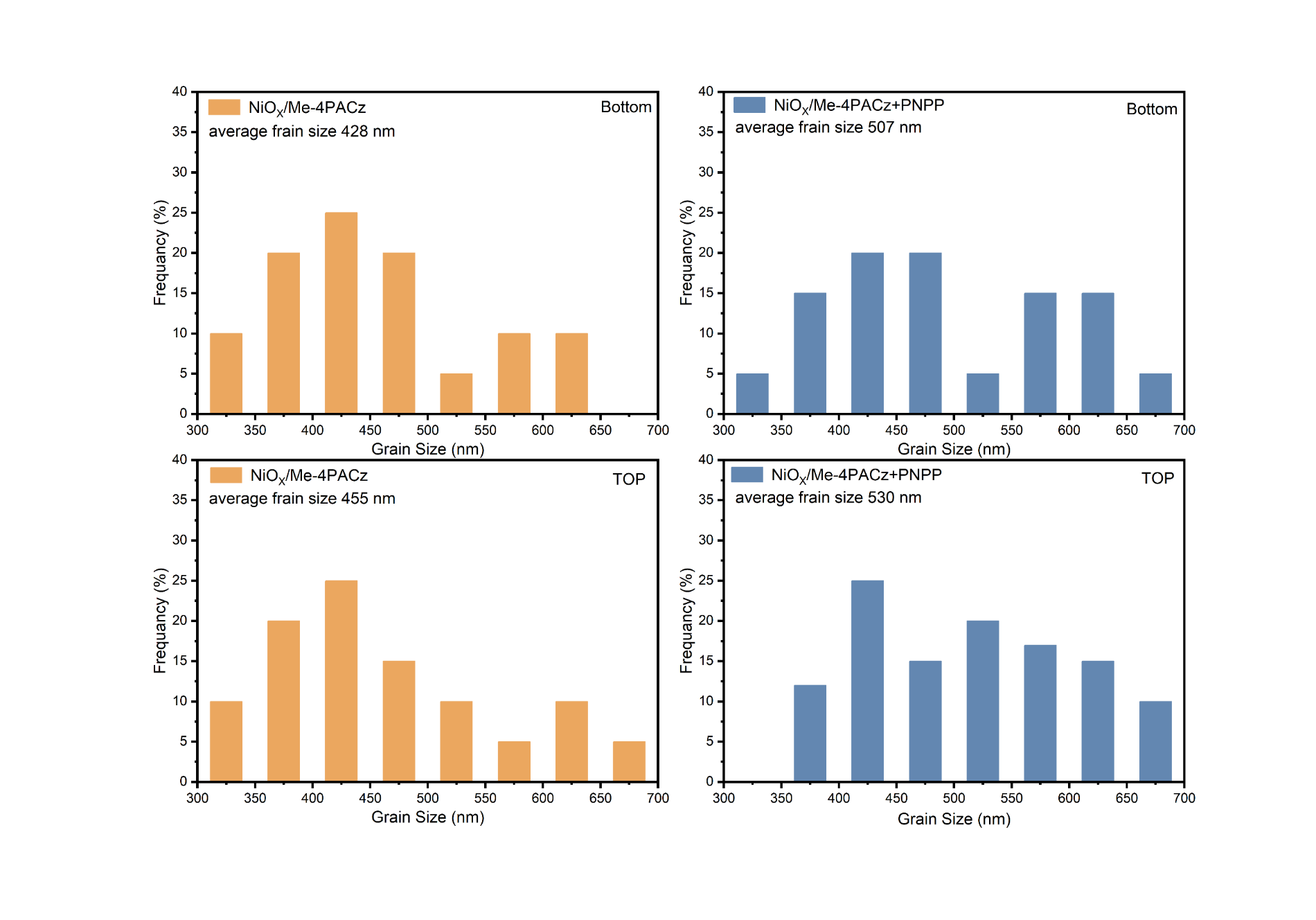


**Figure S13.** Bottom and top corresponding grain size distributions of the NiO_x_/Me-4PACz and NiO_x_/Me-4PACz+PNPP.


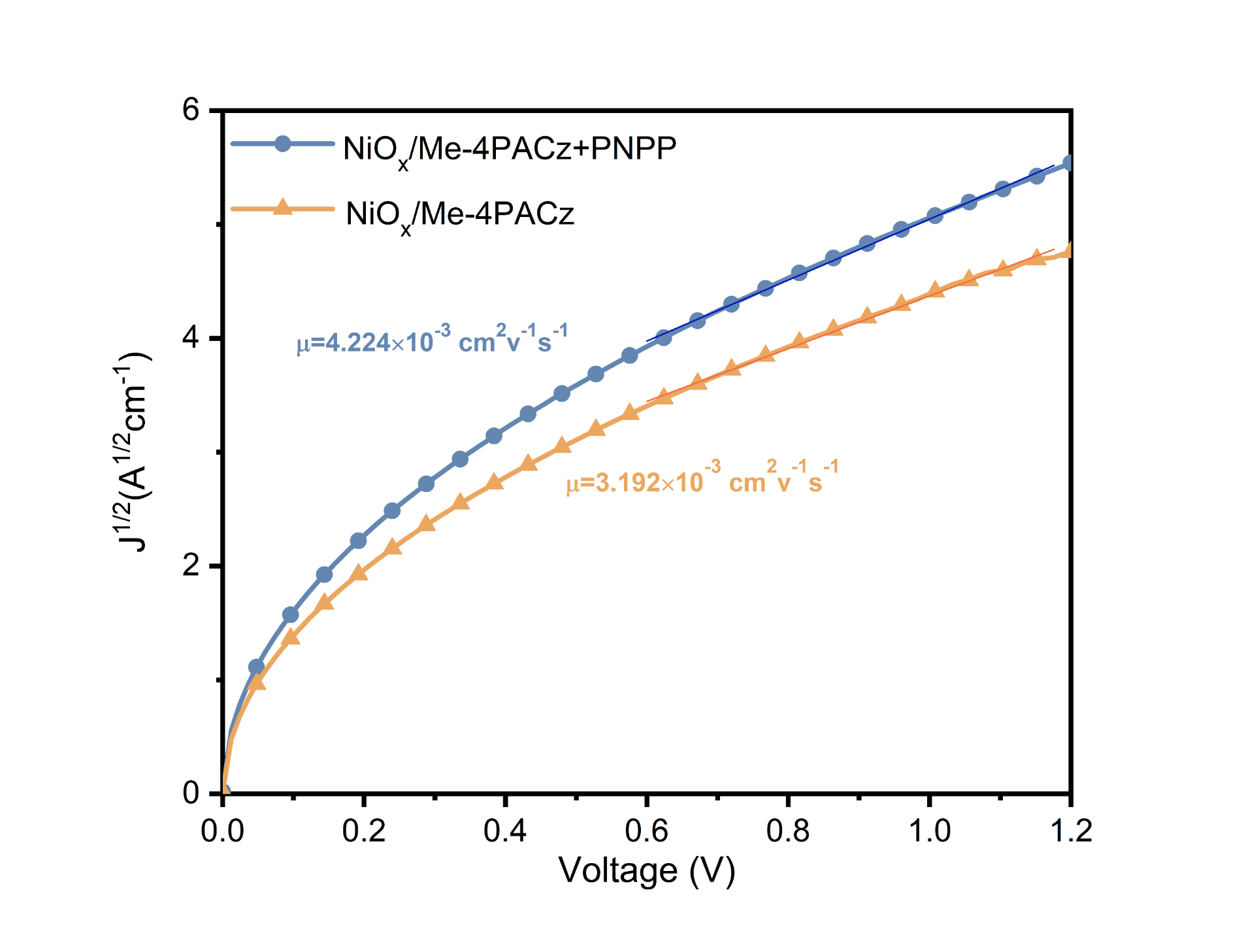


**Figure S14.** *J*–*V* curves of the devices for evaluating the hole mobility of the NiO_x_/Me-4PACz+PNPP and NiO_x_/Me-4PACz.

Note: In calculating hole mobilities, the relative permittivity (εᵣ) was held constant at NiO_x_ reference values throughout.


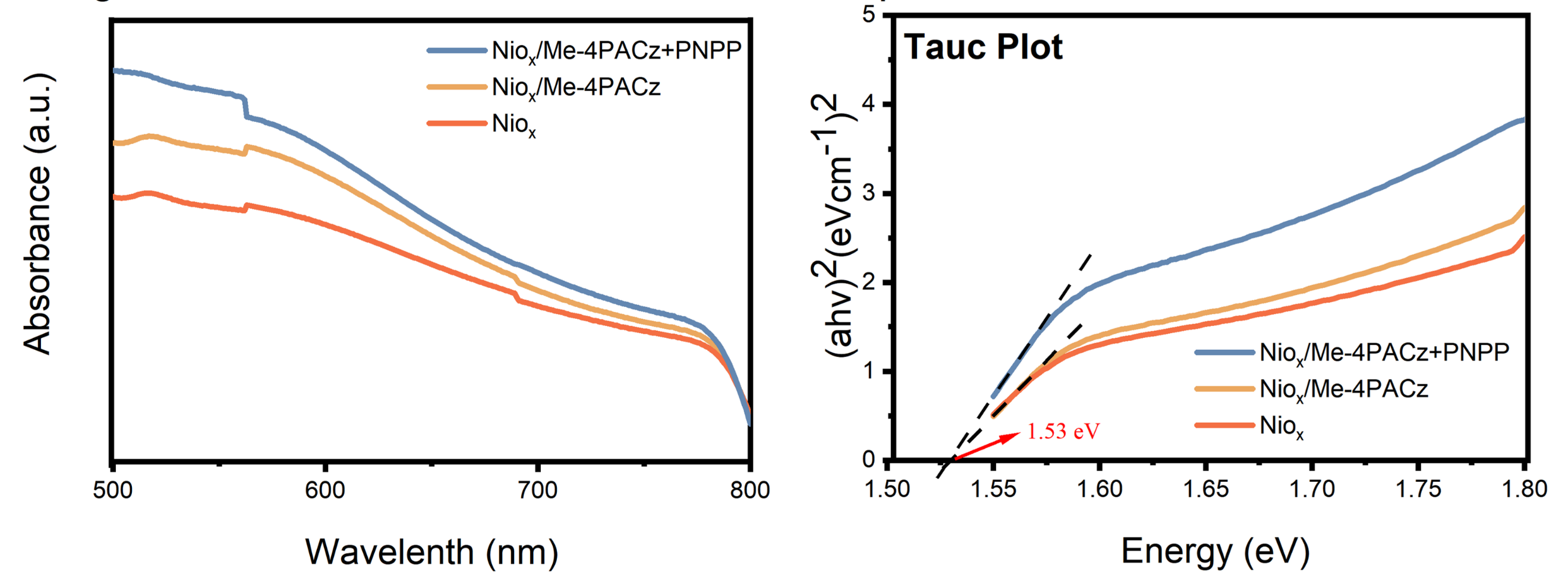


**Figure S15.** Ultraviolet visible (UV-vis) absorption spectra, Tauc Plot of perovskite films deposited on NiO_x_, NiO_x_/Me-4PACz and NiO_x_/Me-4PACz+PNPP.


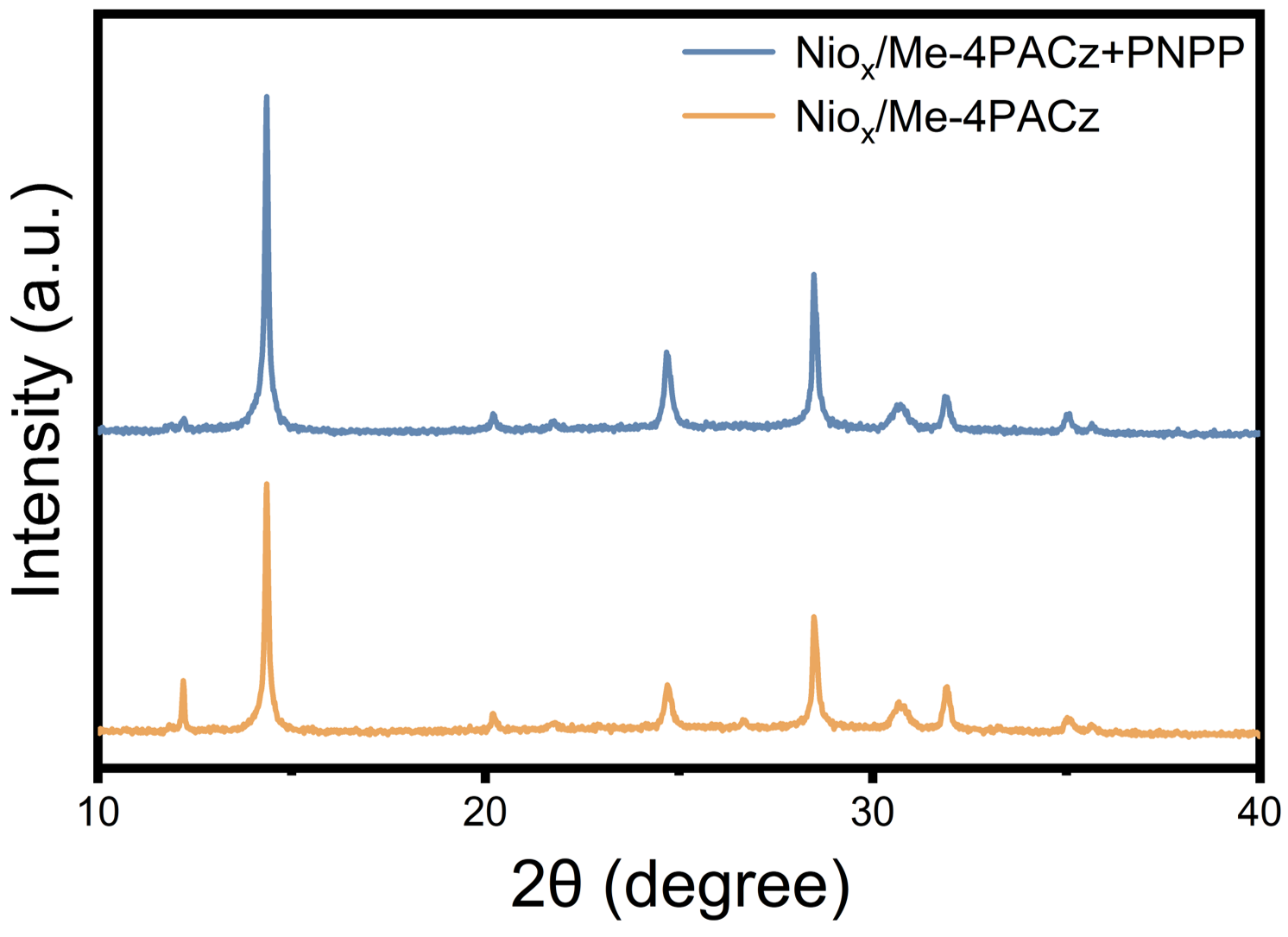


**Figure S16.** XRD patterns of perovskite films deposited on NiO_x_/Me-4PACz and NiO_x_/Me-4PACz+PNPP.


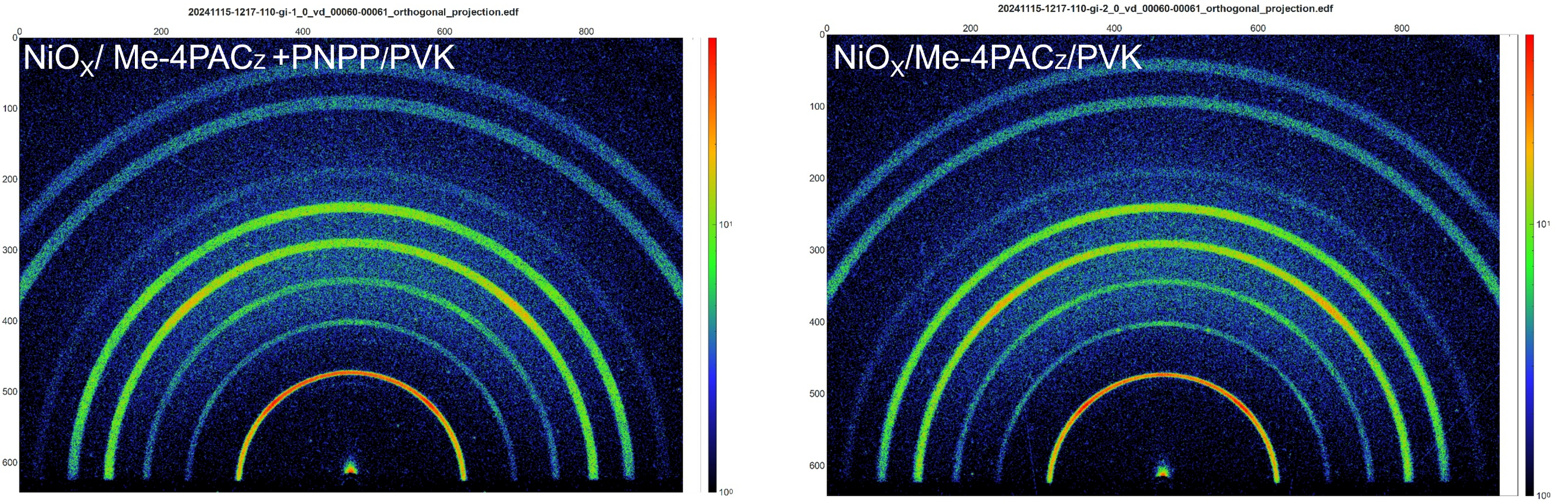


**Figure S17.**  The 2D GIWAXs patterns of perovskite film deposited on NiO_x_/Me-4PACz+PNPP and NiO_x_/Me-4PACz.


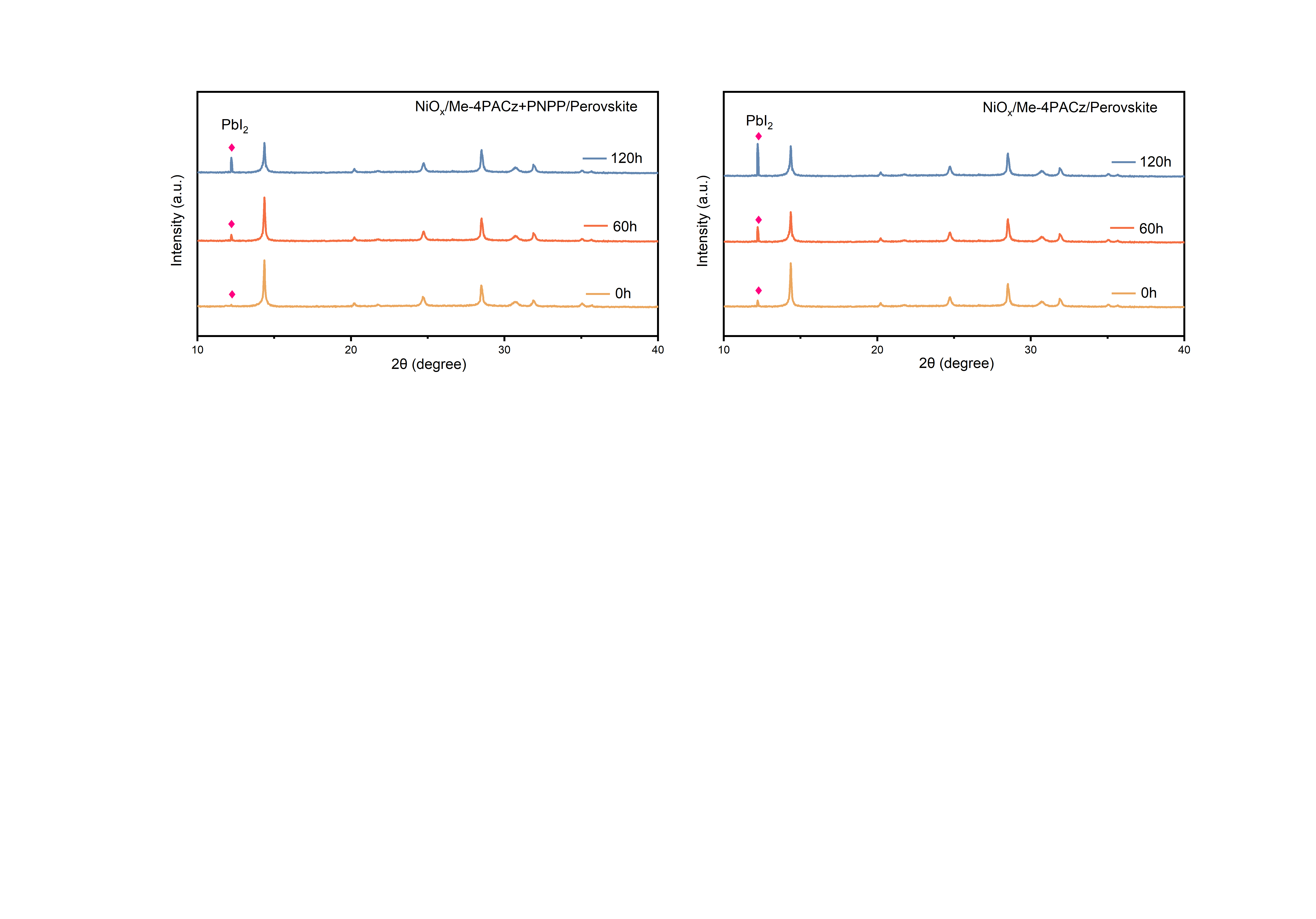


**Figure S18.** Time stability with XRD patterns of NiO_x_/Me-4PACz/perovskite and NiO_x_/ NiO_x_/Me-4PACz+PNPP/perovskite films stored under 25 °C in air atmosphere (30–40% RH).


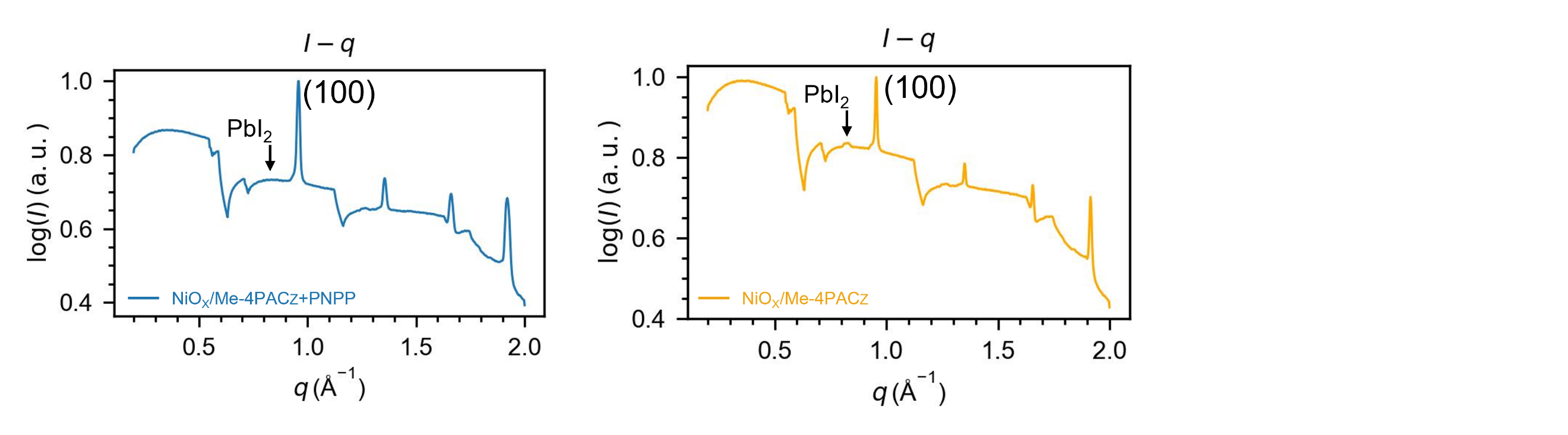


**Figure S19.** Line-cut profiles from GIWAXs patterns of different perovskite films.


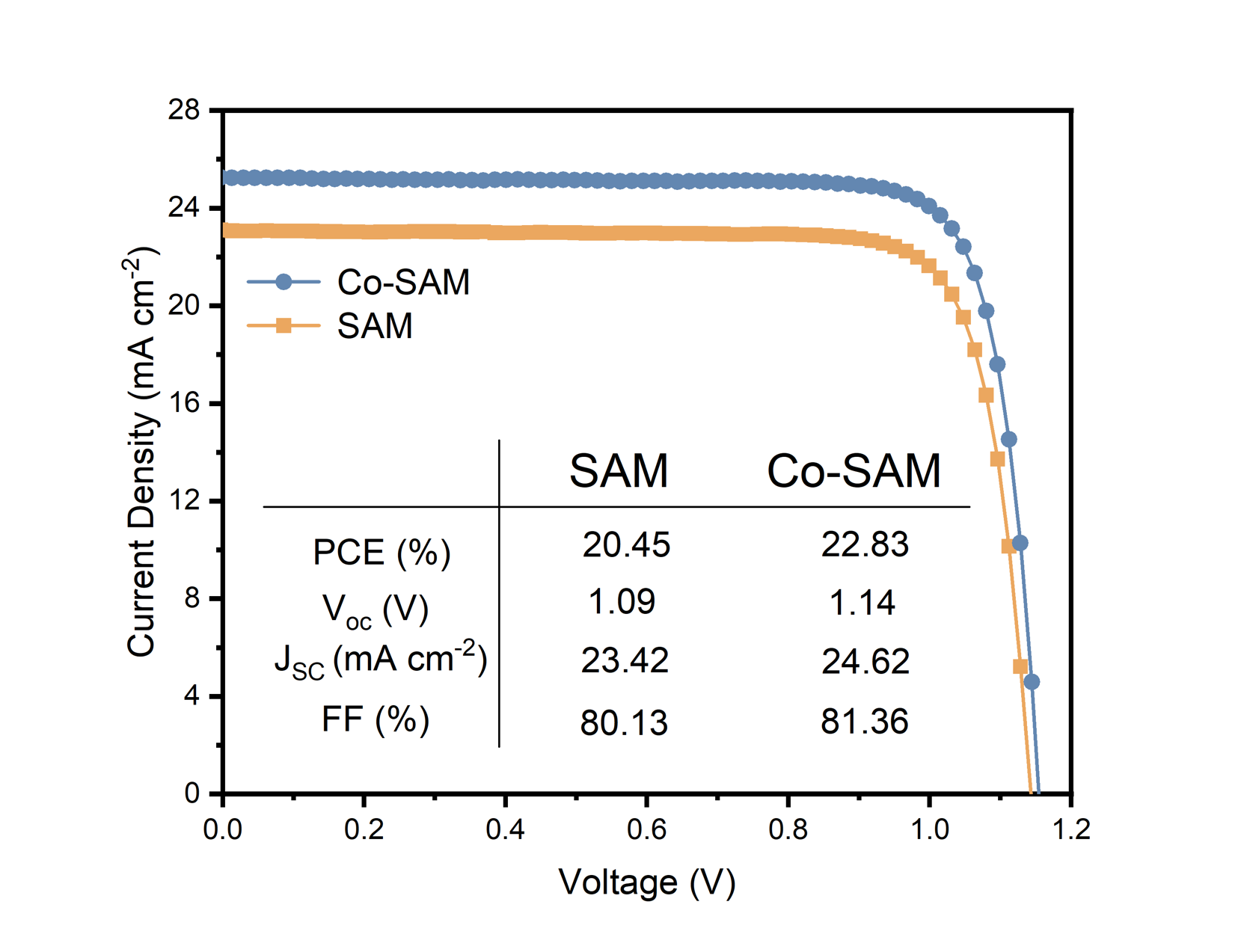


**Figure S20.** *J*–*V* curves of champion SAM and Co-SAM F-PSCs (forward scan).

**
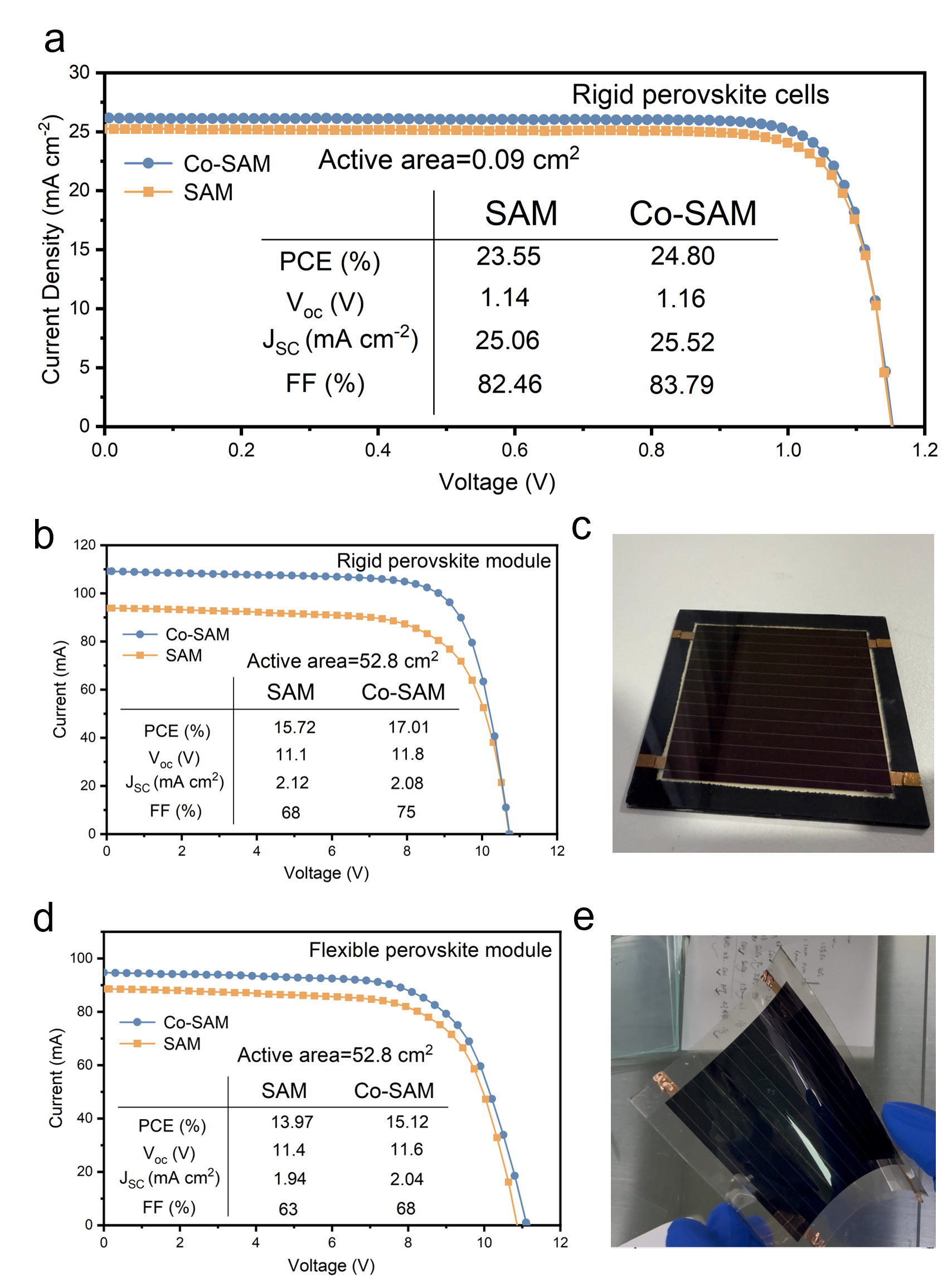
**

**Figure S21.** (a) *J*–*V* curves of champion small-area PSCs fabricated with single SAM and Co-SAM interfacial layers. (b) *J*–*V* curves of champion large-area PSCs fabricated with single SAM and Co-SAM.(c) Photographic image of large-area PSCs. (d) *J*–*V* curves of champion large-area F-PSCs fabricated with single SAM and Co-SAM.(e) Photographic image of large-area F-PSCs.


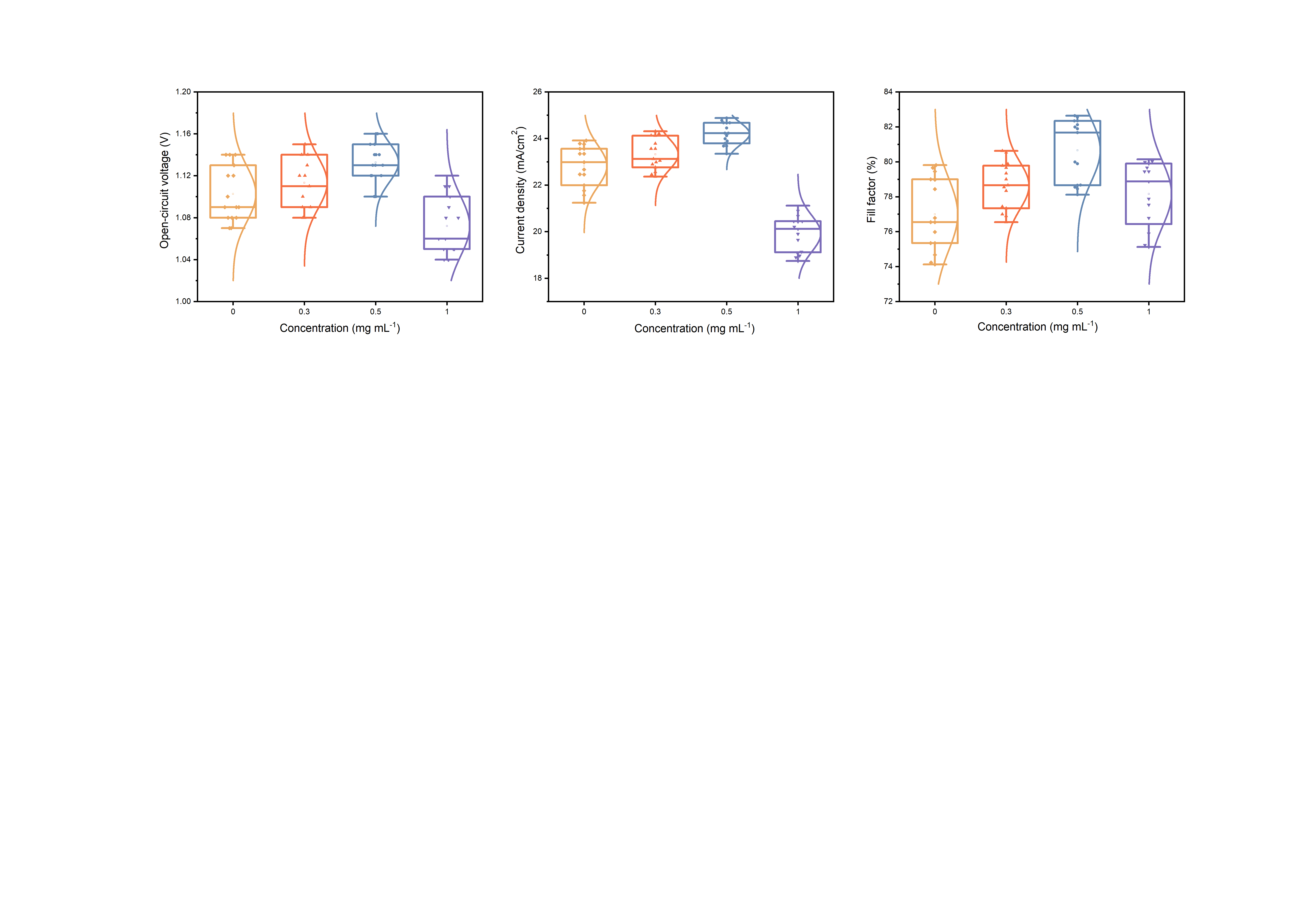


**Figure S22.** Distribution of *V*oc, *J*sc, and FF parameters in *J*–*V* characterization from 15 cells of each kind of device.

**
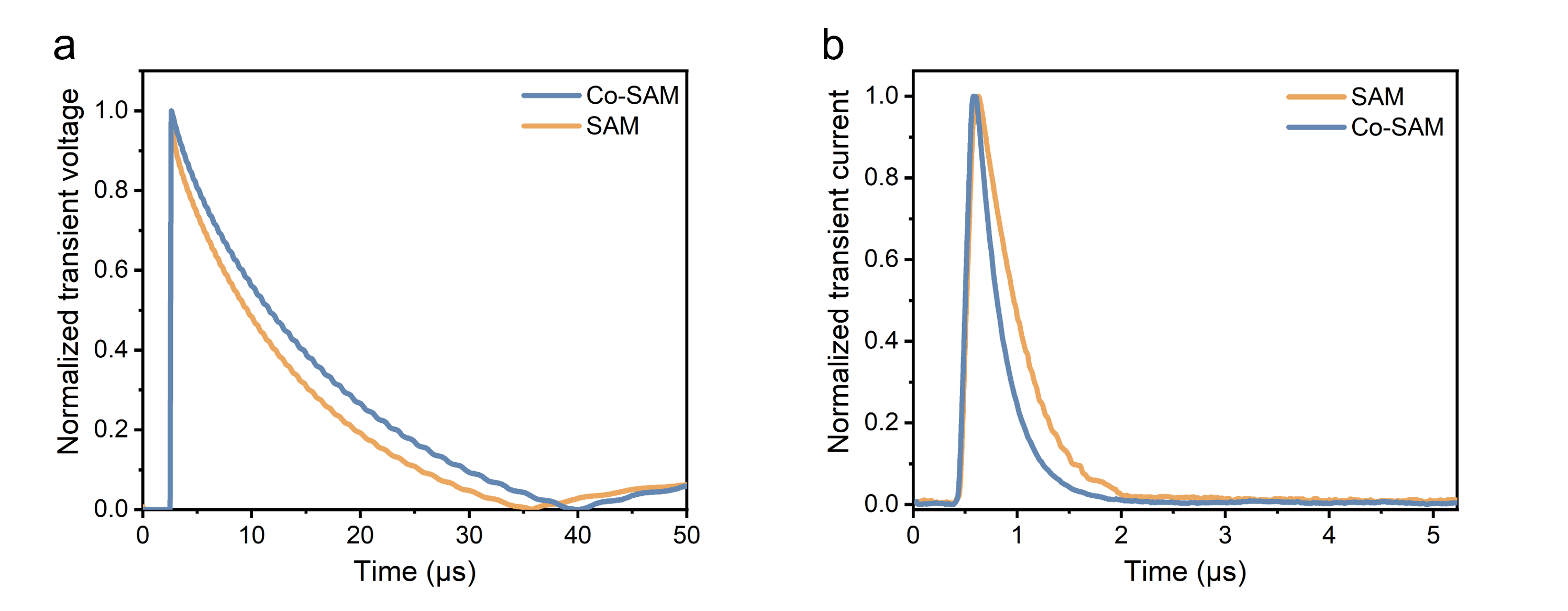
**

**Figure S23.** (a) TPV measurements of devices modified with Co-SAM and SAM.(b) TPC measurements of devices modified with Co-SAM and SAM.

**
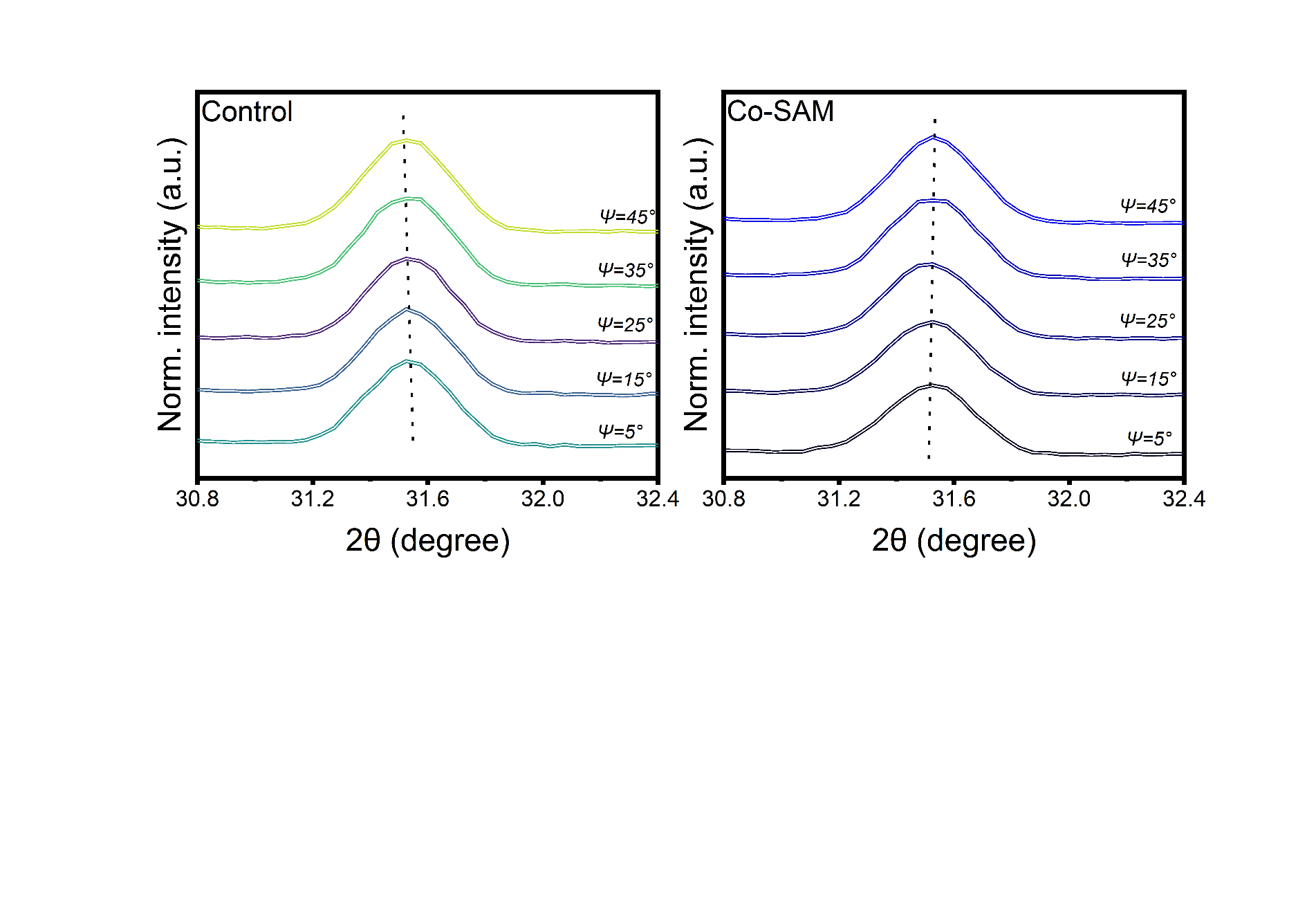
**

**Figure S24.** GIXRD with different instrumental values (0°-45°) for perovskite film deposited on NiO_x_/Me-4PACz and NiO_x_/Me-4PACz+PNPP.

**
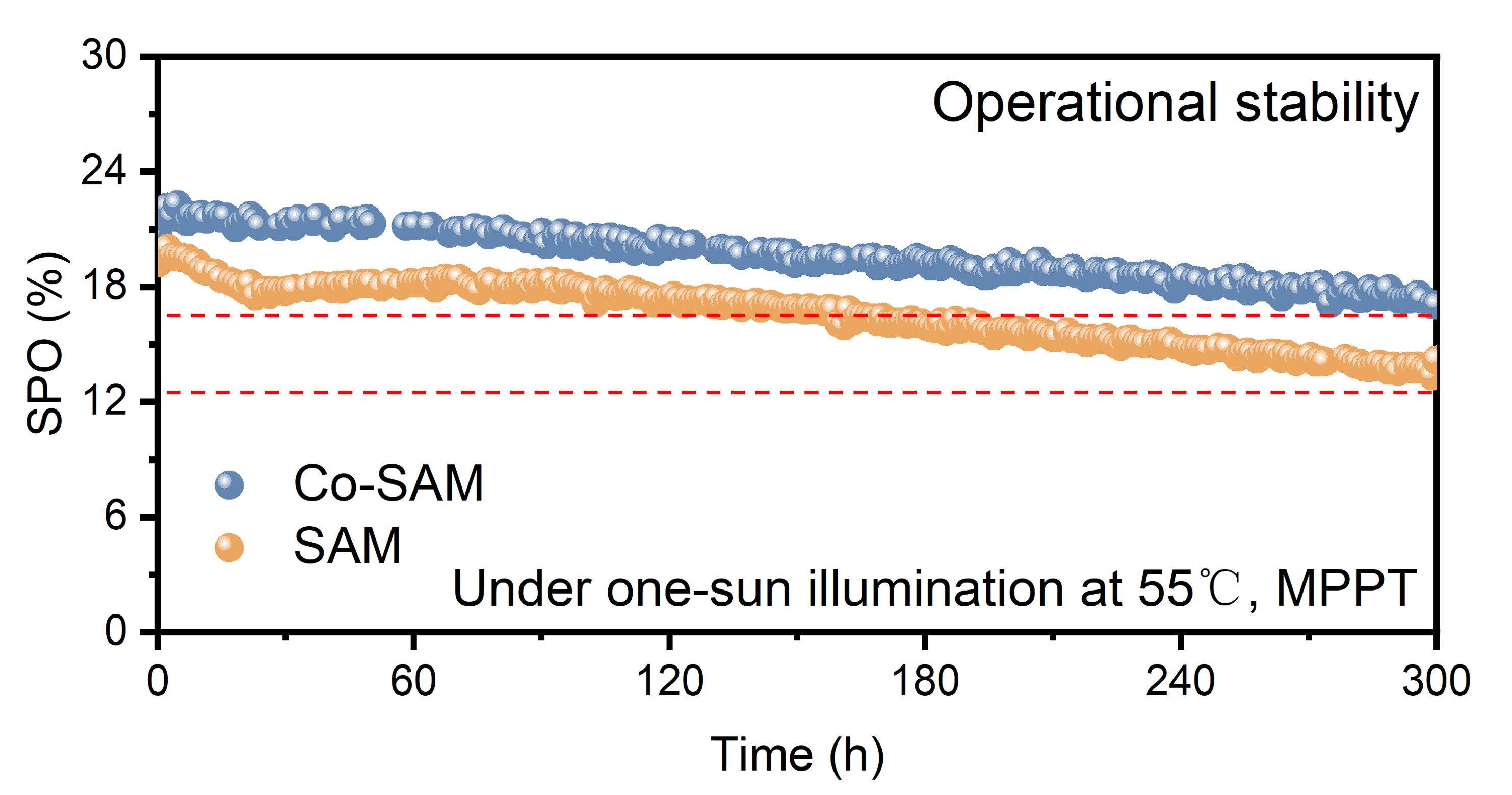
**

**Figure S25.** SPO of the unencapsulated F-PSCs measured at MPP under continuous one-sun illumination.

**
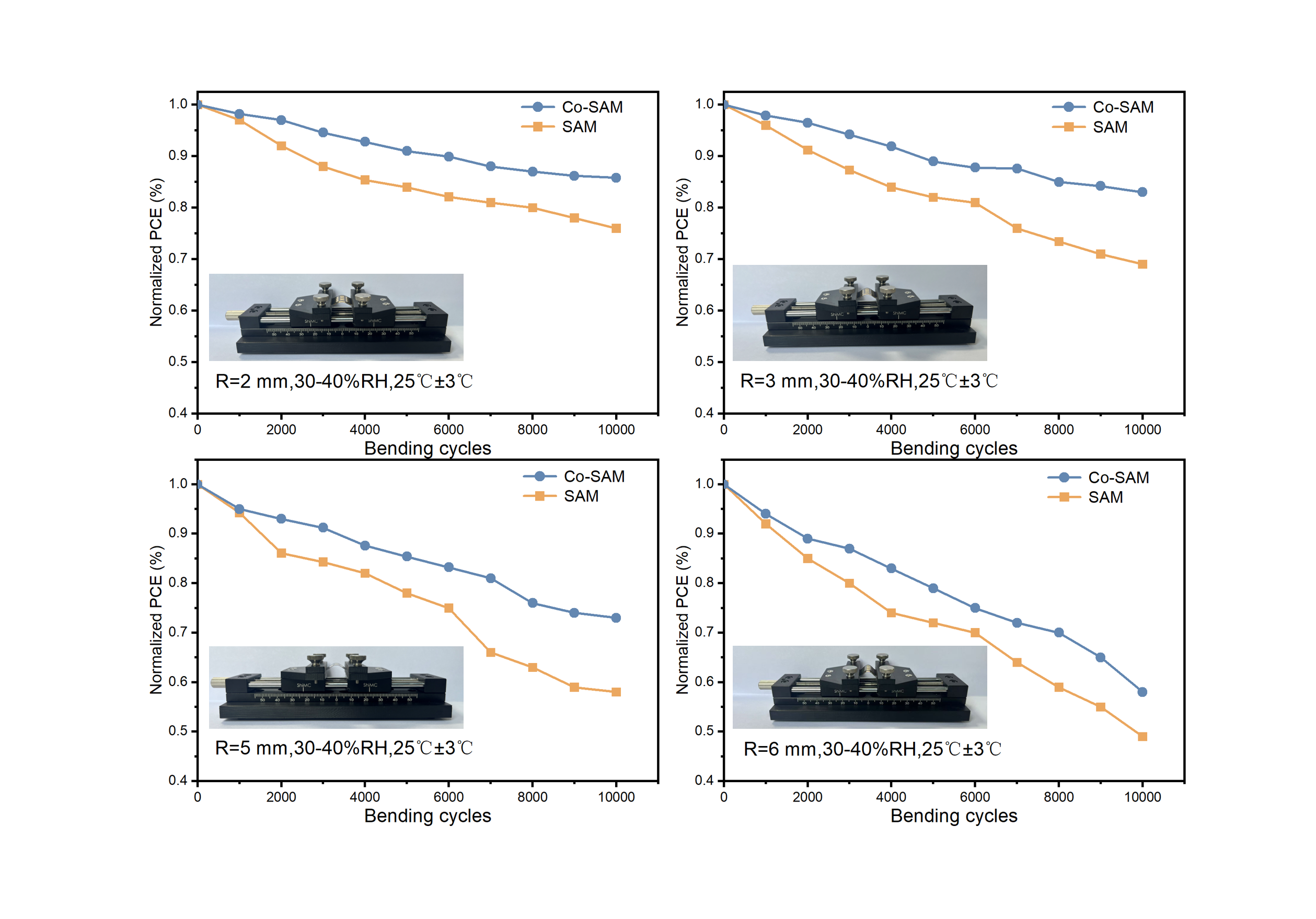
**

**Figure S26.** The evolution of device efficiency under repeated bending at radii of 2 mm, 3 mm, 5 mm, and 6 mm.

**
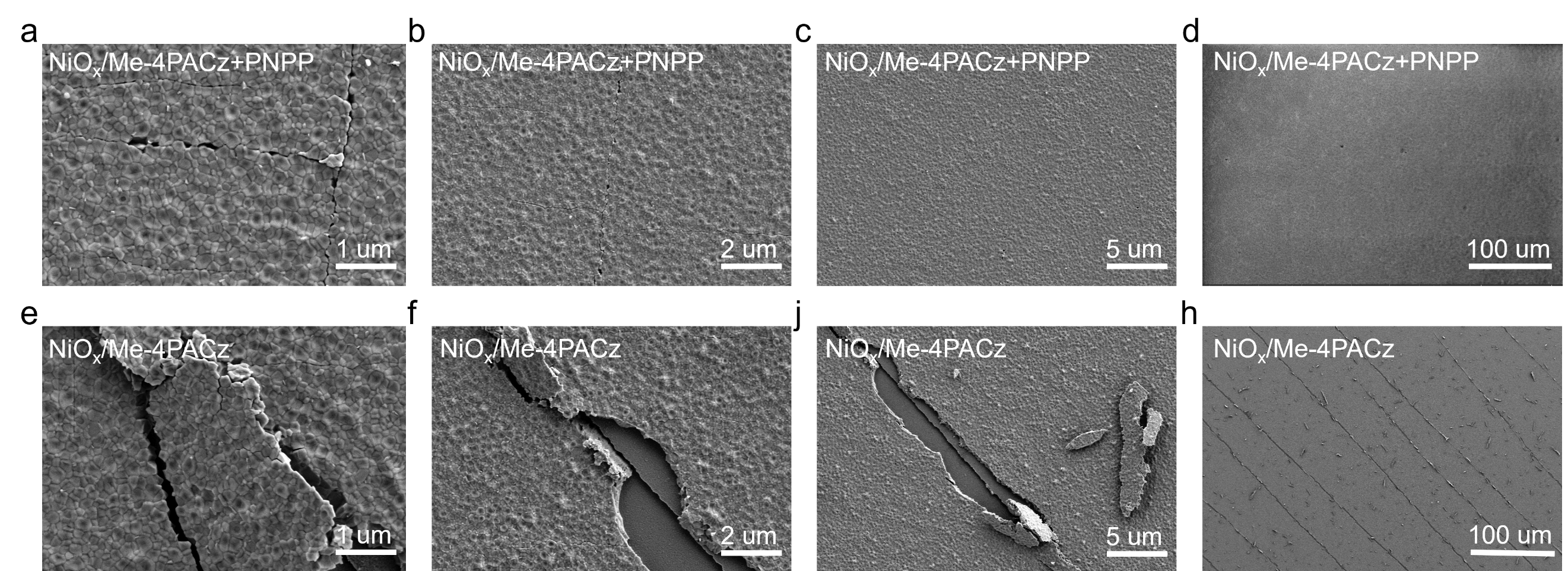
**

**Figure S27.** (a-d) SEM images after 6000 bending cycles of NiO_x_/Me-4PACz+PNPP. (e-h) SEM images after 6000 bending cycles of NiO_x_/Me-4PACz.

**
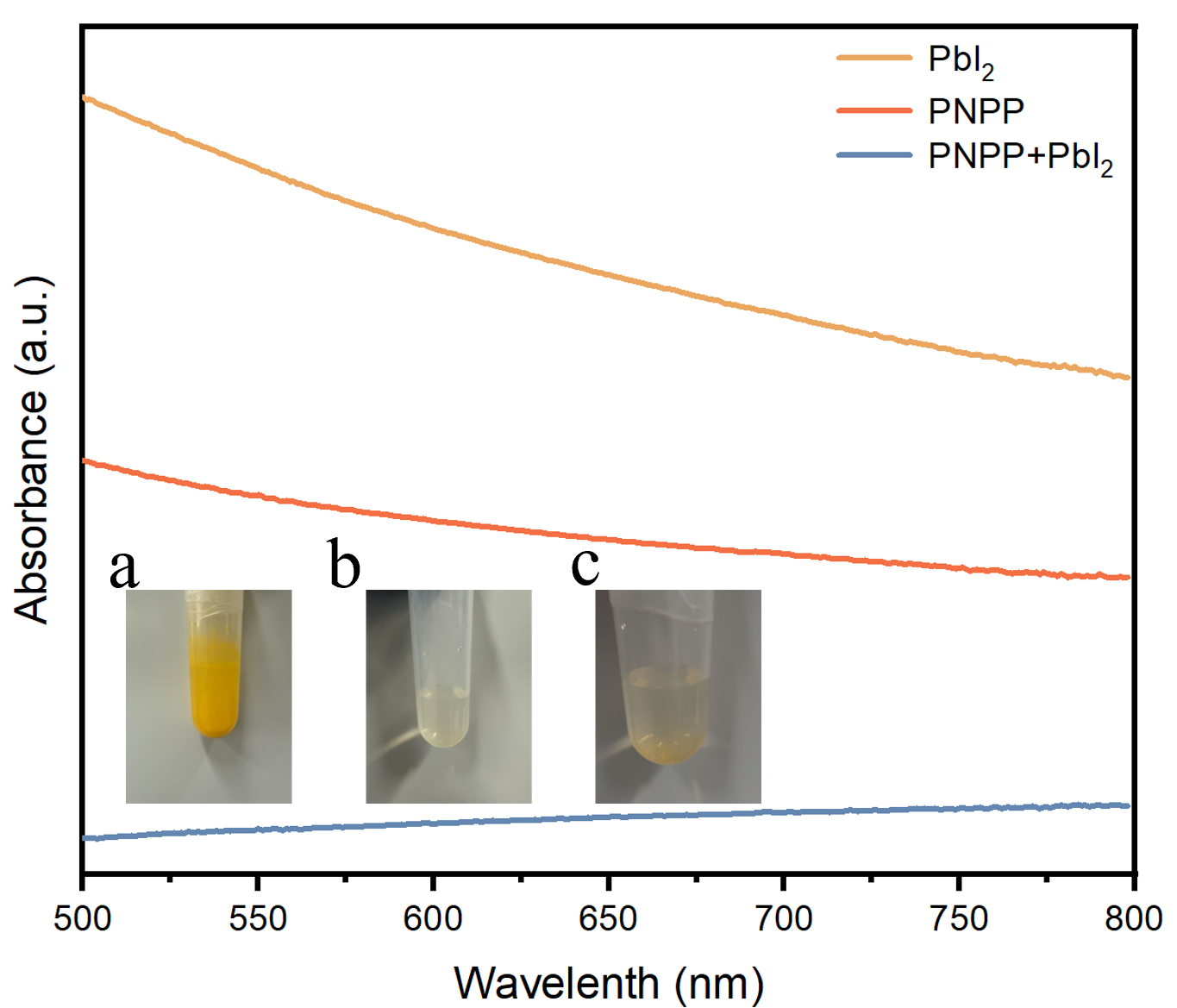
**

**Figure S28.** The UV-Vis absorption intensity of PbI₂/H₂O, PNPP/H₂O and PNPP+PbI₂/H₂O. a. PbI_2_/H_2_O b. The supernatant was collected after PbI_2_/H_2_O was left to stand for 12 hours c. PNPP was added to the supernatant.


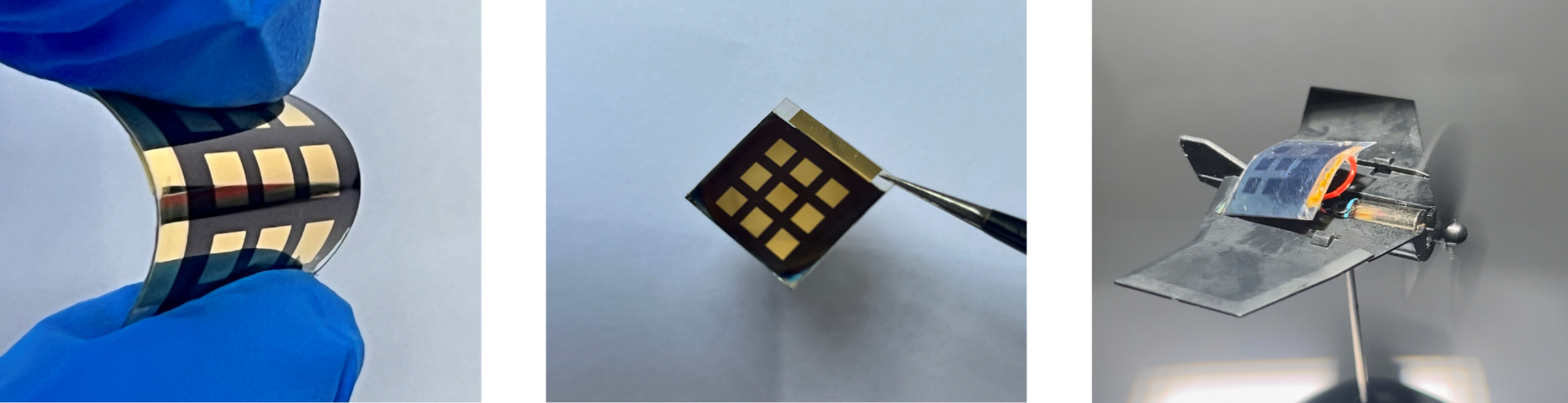


**Figure S29.** Device physical diagram and perovskite solar cell airplane model.

**
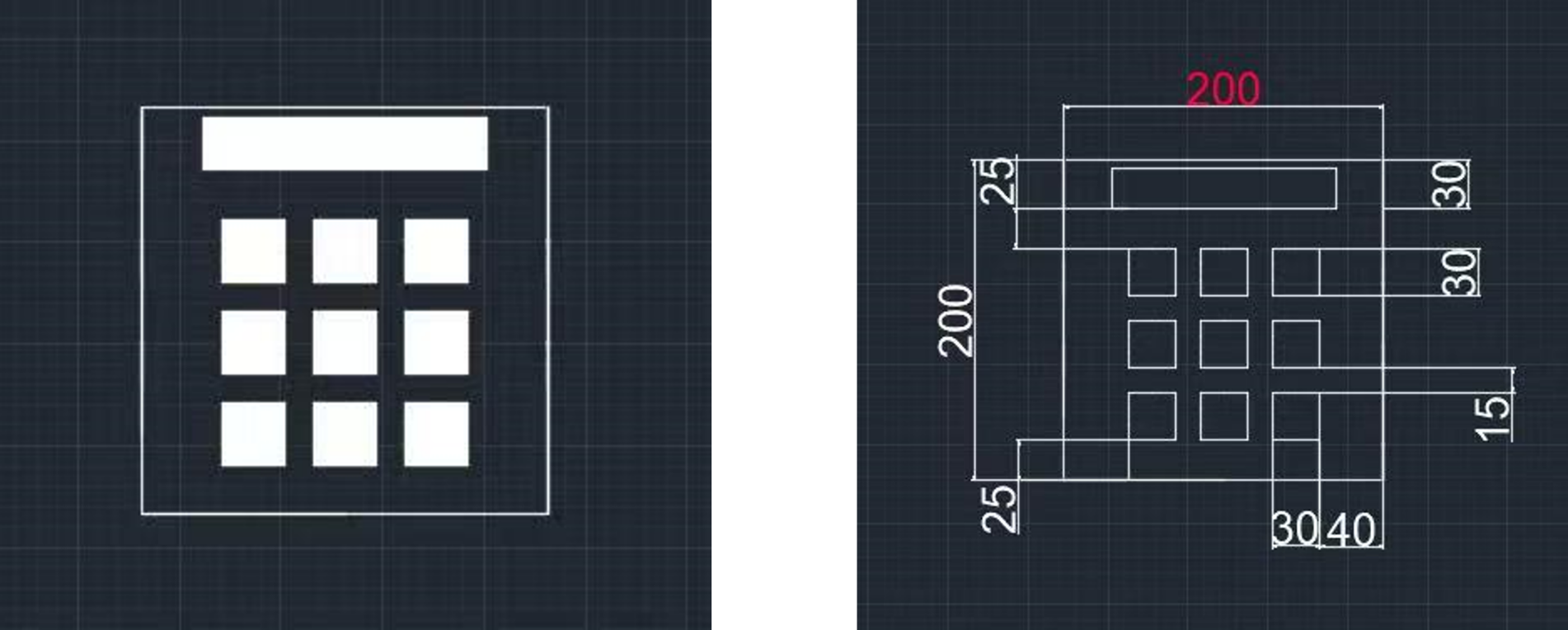
**

**Figure S30.** The mask with the detailed sizes that is used for the metal top electrode.

**References**

[1] Q. Sun, X. Meng, G. Liu, S. Duan, D. Hu, B. Shen, B. Kang, S. R. P. Silva, *Adv. Funct. Mater.* **2024**, 34.

[2] P. Liu, H. Wang, T. Niu, L. Yin, Y. Du, L. Lang, Z. Zhang, Y. Tu, X. Liu, X. Chen, S. Wang, N. Wu, R. Qin, L. Wang, S. Yang, C. Zhang, X. Pan, S. Liu, K. Zhao,  *Energy Environ. Sci.* **2024**, 17, 7069.

[3] J. Chen, X. Fan, J. Wang, J. Wang, J. Zeng, Z. Zhang, J. Li, W. Song, *Acs Nano* **2024**, 18, 19190.

[4] X. Chen, W. Cai, T. Niu, H. Wang, C. Liu, Z. Zhang, Y. Du, S. Wang, Y. Cao, P. Liu, W. Huang, C. Ma, B. Yang, S. Liu, K. Zhao, *Energy Environ. Sci.* **2024**, 17, 6256.

[5] Y. Wang, R. Cao, Y. Meng, B. Han, R. Tian, X. Lu, Z. Song, S. Yang, C. Lu, C. Liu, Z. Ge, *SCI CHINA CHEM.* **2024**, 67, 2670.

[6] Y. Wang, Y. Meng, C. Liu, R. Cao, B. Han, L. Xie, R. Tian, X. Lu, Z. Song, J. Li, S. Yang, C. Lu, Z. Ge, *Joule.* **2024**, 8, 1120.
